# Osteogenic Differentiation Triggered by Intracellular Magnetoelectric Stimulation of Core–Shell Nanotransducers under Remotely Applied Magnetic Fields

**DOI:** 10.1021/acsnano.5c10509

**Published:** 2025-12-05

**Authors:** Maria C. Mendes, Elisa A. G. Martins, Roman V. Chernozem, Polina V. Chernozem, Catarina C. Custódio, Roman A. Surmenev, Andrei L. Kholkin, Ana S. Silva, João F. Mano

**Affiliations:** † Departament of Chemistry, CICECO, Campus Universitário de Santiago, University of Aveiro, Aveiro 3810-193, Portugal; #Physical Materials Science and Composite Materials Centre, ‡International Research & Development Center “Piezo- and Magnetoelectric Materials”, Research School of Chemistry & Applied Biomedical Sciences, 65078 National Research Tomsk Polytechnic University, Tomsk 634050, Russia; § Department of Physics & CICECO, Campus Universitário de Santiago, University of Aveiro, Aveiro 3810-193, Portugal

**Keywords:** core−shell nanoparticles, magnetoelectric stimulation, static and cyclic magnetic fields, 3D spheroids, osteogenic differentiation

## Abstract

Magnetoelectric nanoparticles (MENPs), combining a magnetostrictive core with a piezoelectric shell, offer a promising route for remote-controlled biomedical applications by converting external magnetic fields into electric cues. However, the clinical translation of these materials remains limited due to the toxicity of high-performance piezoelectric materials, which typically contain lead. Previously, we developed lead-free MENPs comprising manganese ferrite oxide (MFO) core nanoparticles (NPs) coated with a Ba_0.85_Ca_0.15_Zr_0_._1_Ti_0.9_O_3_ (BCZT) piezoelectric shell (MFO@BCZT). While these nanotransducers exhibit robust magnetic responsiveness and piezoelectric performance comparable to lead-based ceramics, their role in producing *in situ* electrical cues to accelerate bone repair remains unexplored. Given the established role of electrical stimulation in bone remodeling, this study explores the potential of MFO@BCZT MENPs to promote the osteogenic differentiation of human adipose-derived stem cells (hASCs) after internalization, assembly into magnetized 3D spheroids, and subsequent embedding in gelatin methacryloyl hydrogels, to better recapitulate physiologically relevant microenvironments. Differentiation was assessed under static and cyclic magnetic field (CMF) conditions and compared to spheroids containing bare MFO NPs and spheroids without NPs. Results revealed that MFO and MFO@BCZT NPs were cytocompatible; however, MFO@BCZT MENPs significantly enhanced osteogenic marker expression and mineral deposition compared to both controls, with CMF further amplifying these effects. Under CMF stimulation, MFO@BCZT MENPs produced a mineralized matrix with a calcium-to-phosphorus molar ratio of 1.67, aligning precisely with native bone apatite. Overall, by restoring the bioelectric properties of bone at the target region, this study positions MFO@BCZT MENPs as a compelling platform for future smart bone therapies.

## Graphical abstract

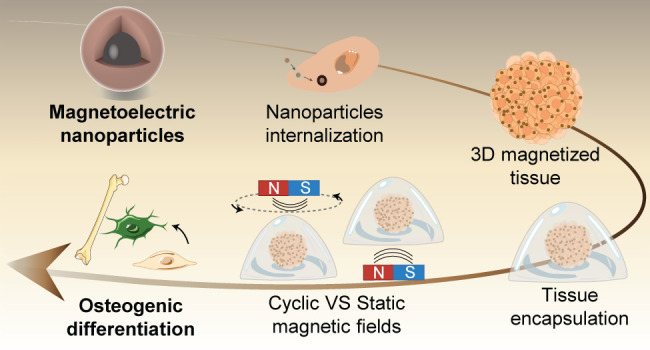

Bone tissue is an essential part of the human body, providing structural support, protecting internal organs, and serving as a reservoir for essential minerals. Beyond its mechanical role, bone possesses intrinsic bioelectrical properties, including dielectric, piezoelectric, pyroelectric, and ferroelectric properties, which all play a central role in its development, remodeling, and fracture healing.^1^ While these properties endow bone with a capacity for self-repair, critical-sized defects exceed this regenerative potential, requiring clinical intervention for proper repair.^2–4^

Common treatment strategies include bone fixation implants, autografts, allografts, and electrical stimulating therapies.^2–6^ However, each approach presents limitations: fixation devices may necessitate secondary surgeries; autografts can lead to donor-site morbidity; and allografts risk immune rejection and disease transmission.^2^ Electrical stimulation therapies, which involve the implantation of electrodes percutaneously or transcutaneously at the defect site and rely on an external power source or electromagnetic coil to deliver the appropriate electrical signal over time, introduce additional risks such as infection, skin irritation and thermal injury, electrical shock, and concerns regarding device biocompatibility and battery disposal.^3,7^

Understanding bone physiology has been relevant to develop solutions for bone repair, in particular for bone regeneration.^8^ Inspired by the electrophysiological properties of native bone, piezoelectric materials have attracted growing interest in biomedical applications due to their ability to deliver electrical stimuli to cells in response to applied mechanical stress (direct piezoelectricity).^9,10^ This unique property enables the creation of self-powered electrical systems favorable for clinical use, without the need for electrode implantation and any external electrical power source.^7,11^ Despite these advantages, the development of functional piezoelectric materials for biomedical applications faces two main challenges, namely the mechanical stimuli required to activate the piezoelectric effect and the materials composition required to ensure biocompatibility, biodegradability, and a robust piezoelectric response.^7,9,12^

Although body motion can activate piezoelectric materials, physical activity is often limited postoperatively, failing to generate stress for piezoelectric responses.^7–12^ As an alternative, low-powered ultrasounds can be used to stimulate the piezoelectric materials and generate electrical signals.^9^ While ultrasounds offer a noninvasive approach with adjustable frequencies to minimize cell damage, ultrasound waves are subjected to refraction across tissues of varying densities, scattering at tissue interfaces, reflection, and absorption, ultimately leading to heat generation, which may be harmful to tissues with prolonged exposure.^7,11,13^

To overcome these challenges, magnetoelectric (ME) composite nanomaterials have emerged as a promising strategy for electrical stimulation, as they generate electrical charges in response to external magnetic fields (direct magnetoelectric effect), which, unlike ultrasound, penetrate deeply into the body without attenuation.^7,14–17^ ME materials are typically composed of a ferromagnetic and piezoelectric phase coupled elastically.^17–19^ Under a low-frequency magnetic field, the ferromagnetic phase undergoes magnetostrictive strain, which is transferred to the piezoelectric phase, producing a localized electric field.^20^ The concept of ME nanoparticles (NPs) (MENPs) was first introduced by Yue et al. in 2012 as a noninvasive brain stimulation method capable of crossing the blood–brain barrier (BBB) and restoring neuronal communication in Parkinson’s disease.^21^ Subsequent *in vitro*, *ex vivo*, and *in vivo* studies validated their safety, BBB penetration, magnetic targeting, and ability to trigger cellular biophysical and biochemical processes.^14,15,18,22–24^ Importantly, MENPs, typically smaller than 30 nm, have shown no cytotoxic effects in the brain or major organs (kidneys, lungs, liver, spleen), nor do they impair hepatic, renal, or neurobehavioral functions.^25,26^ Their small size also enables rapid clearance, further supporting their potential as safe and effective nanotherapeutics.^26^

Core–shell nanostructures represent one of the most extensively studied MENP configurations to date. Many reported systems use Fe_3_O_4_ NPs as the magnetostrictive core, as it is the only metal iron NPs approved by the FDA, and explore doping with Co^2+^, Ni^2+^, or Mn^2+^ to improve stability and performance, yielding CoFe_2_O_4_, NiFe_2_O_4_, or MnFe_2_O_4_ NPs, respectively.^17,27^ For the piezoelectric shell, a key design criterion is the d_33_ coefficient, which quantifies the amount of induced charge per unit of mechanical stress.^28^ While lead-based ceramics such as PZT (*d*_33_ = 300–1000 pC/N) and PMN–PT (*d*_33_ ≥ 800 pC/N) exhibit exceptional performance, their toxicity precludes biomedical use.^3,17^ Biocompatible candidates include barium titanate (BaTiO_3_, BT, *d*_33_ = 190 pC/N) and its derivatives like sodium bismuth titanate (NBT, *d*_33_ = 100–400 pC/N), as well as polyvinylidene fluoride (PVDF, *d*_33_ = 22 pC/N) derivatives, though they exhibit lower d_33_ values, which result in a reduced piezoelectric response.^29,30^

To overcome this challenge, we have previously developed lead-free MENPs composed of MnFe_2_O_4_ (MFO) cores coated with a Ba_0.85_Ca_0.15_Zr_0.1_Ti_0.9_O_3_ (BCZT) piezoelectric shell (MFO@BCZT).^31^ MFO is a soft magnetic material characterized by high magnetic permeability, low coercivity, and high chemical stability. Its high permeability enables rapid magnetization even under low-intensity magnetic fields, while low coercivity ensures easy and reversible demagnetization. Mn^2+^ doping enhances chemical stability and biocompatibility due to the body’s natural manganese metabolism.^32–34^ Meanwhile, the inclusion of BCZT provides a biocompatible shell with a d_33_ value of 620 pC/N, which is one of the highest coupling coefficients for lead-free piezoelectric materials available today, offering strong piezoelectric performance with excellent cytocompatibility.^28,30^

Leveraging the synergy between MFO and BCZT, we hypothesized that MFO@BCZT MENPs can significantly enhance mechanical sensitivity and ME conversion under low intensity magnetic fields, supporting bone regeneration via *in situ* electrical stimulation. To closely replicate the *in vivo* environment, MFO@BCZT MENPs were internalized into human adipose-derived stem cells (hASCs) and subsequently assembled into 3D spheroids. Outcomes were assessed under static and cyclic magnetic field (CMF) exposure and compared to spheroids containing bare MFO NPs lacking the piezoelectric shell and spheroids devoid of magnetic functionality, demonstrating the superior performance of MFO@BCZT MENPs in promoting osteogenic differentiation via intracellular ME stimulation.

## Results and Discussion

Given the established role of magnetic fields in inducing osteogenesis, this work investigates the potential of MFO@BCZT MENPs to enhance the osteogenic differentiation of hASC spheroids under static magnetic field (SMF) and CMF conditions.^35–39^ The hASCs were chosen as the cell model because of their well-characterized osteogenic differentiation potential, low immunogenicity, and high availability, as they can be minimally and invasively harvested from adipose tissue discarded during liposuction or abdominoplasty procedures.^40,41^ The results are compared against MFO NPs lacking the piezoelectric component and tissues devoid of magnetic functionality.

### Characterization of MFO@BCZT MENPs

The morphology and internal structure of MFO@BCZT MENPs (Figure 1A) were investigated by high-resolution transmission electron microscopy (HRTEM) (Figure 1B). The results revealed the formation of a thin perovskite BCZT shell (∼4 nm) on the surface of the quasi-spherical MFO NPs (∼23 nm). Energy-dispersive X-ray (EDX) mapping confirmed the distribution of metal ions (Ba and Ti) of the BCZT shell on the surface of the MFO cores (Mn and Fe), validating both the elemental composition and relative abundance at the nanoscale surface. It was observed that the BCZT shell was formed along the faces of the MFO cores, following the crystallographic orientation of the underlying spinel structure.^42^ HRTEM analysis revealed crystalline lattice coherence at the interface of the core and shell: (220) with 3.06 Å and (400) with 2.10 Å for the MFO phase (PDF card #73-1964), while (110) with 2.98 Å and (200) with 2.12 Å for the BCZT phase (PDF card #31-174). These observations suggest an epitaxial growth, which is a significant advantage, as it demonstrates that the core–shell architecture maintains crystallographic coherence. Such coherence enables precise control over the nanostructure and facilitates more efficient strain transmission from the magnetostrictive core to the piezoelectric shell owing to the well-defined atomic interface.^42,43^

**Figure 1:**
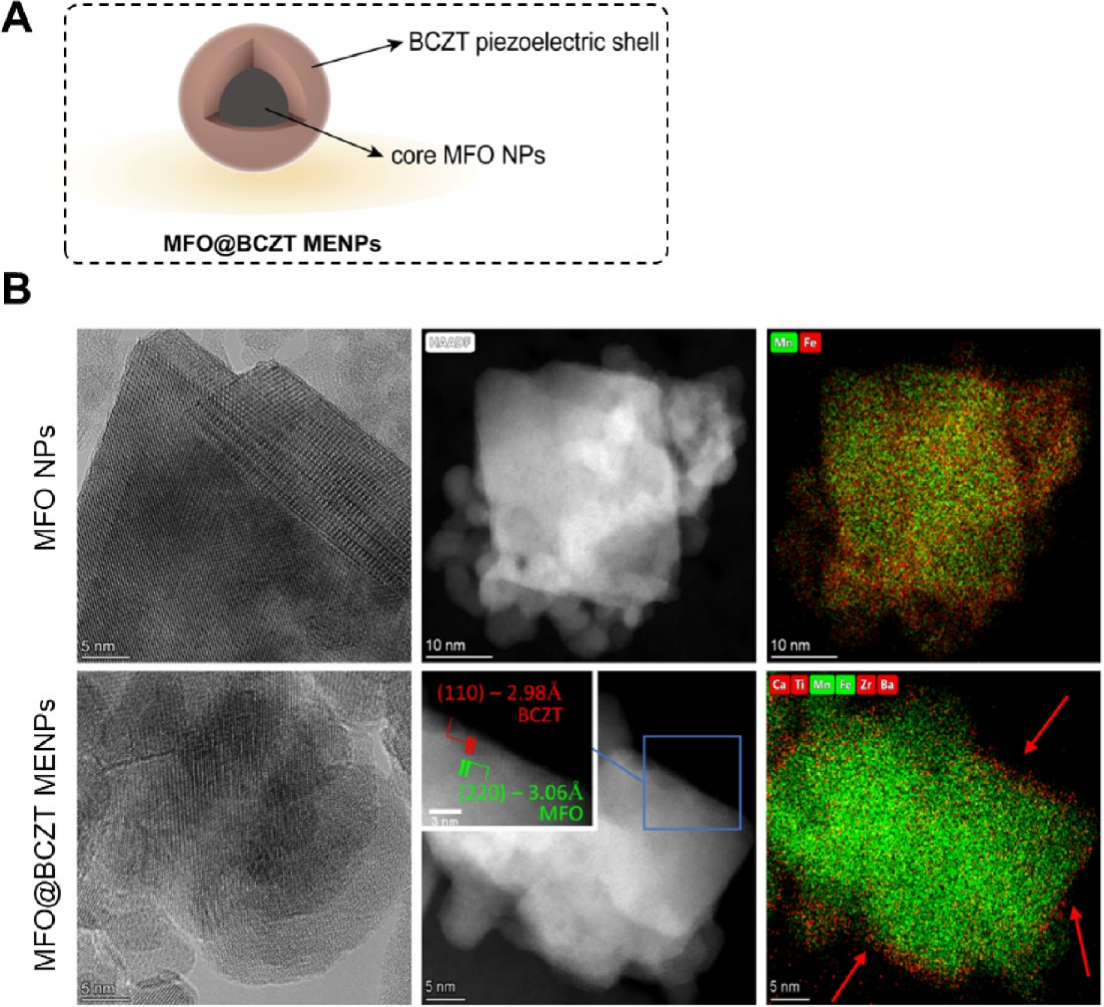
Characterization of the MFO@BCZT MENPs. (A) Schematic illustration of the core–shell structure of the developed MFO@BCZT MENPs. Image created with Adobe Illustrator. (B) High-resolution, high-angle annular dark-field scanning transmission electron microscopy and EDX mapping images for MFO and MFO@BCZT NPs. Red arrows show the growth of the BCZT shell along the faces of the MFO cores.

Figure 2A depicts X-ray diffraction (XRD) patterns for the synthesis of magnetic core MFO NPs and core–shell MFO@BCZT MENPs. The MFO cores exhibited a typical spinel crystalline structure, consistent with the reference pattern from the crystallography open database (COD, COD #10-0319), with no detectable impurities (Table 1). Simultaneously, the successful formation of the perovskite BCZT shell (COD #75-0279) on the surface of citric acid (CA)-functionalized MFO cores was confirmed, without the emergence of other iron oxides. According to the analysis of relative phase composition (Table 1), the phase ratio of MFO to BCZT was 39 to 61% in the core–shell MENPs. Due to the possible coexistence of different noncentrosymmetric crystalline phases with coinciding or close planes, the analysis of the crystalline structure of BCZT by XRD alone is challenging. Therefore, highly sensitive Raman spectroscopy was used to complement the XRD analysis (Figure 2B).^44–48^ As compared to literature data (located at ∼360 and 660 cm^–1^), typical Raman peaks for our MFO NPs were found to be shifted to 320 and 625 cm^–1^.^49^ This effect can be explained by the chelation of citric acid with metal ions.^49^ On the other hand, this effect can also be attested to crystalline structure deformation and differences in NP size.^50,51^ Despite the alterations in MFO NPs after their surface functionalization, Raman spectroscopy uncovered the emergence of ferroelectric BCZT phases in core–shell MENPs (Figure 2B). As displayed in the figure, core–shell MENPs exhibited the presence of five typical peaks for BCZT as follows: 180 [A_1_(LO)], 230 [A_1_(TO)], 286 [A_1_(TO)/B_1_/E­(TO + LO)], 510 [A_1_(TO)], and 725 cm^–1^ [A_1_(LO)/E­(LO)].^45,47,48^ The peaks at 180 and 286 cm^–1^ are attributable to a mixture of orthorhombic and tetragonal phases, while the band at 230 cm^–1^ is related to orthorhombic symmetry.^47^ The detected band at 725 cm^–1^ likely matches the tetragonal phase and a paraelectric cubic phase.^46–48^ Figure 2C shows the X-ray photoelectron spectroscopy (XPS) spectra of the formed MFO core NPs and core–shell MFO@BCZT MENPs. As noted, elements expected for MFO (Mn 2p, Fe 2p, O 1s) and BCZT (Ba 3d, Ca 2p, Zr 3d, Ti 2p, O 1s) were registered without impurities. All other unassigned peaks correspond to typical Auger transitions, resulting from the emission of Auger electrons from atoms, or other lines (e.g., 1s and 2s) associated with the elements. The presence of C 1s may be associated with typical adventitious carbon and citric acid used for the surface functionalization of core NPs and core–shell MENPs.

**Figure 2:**
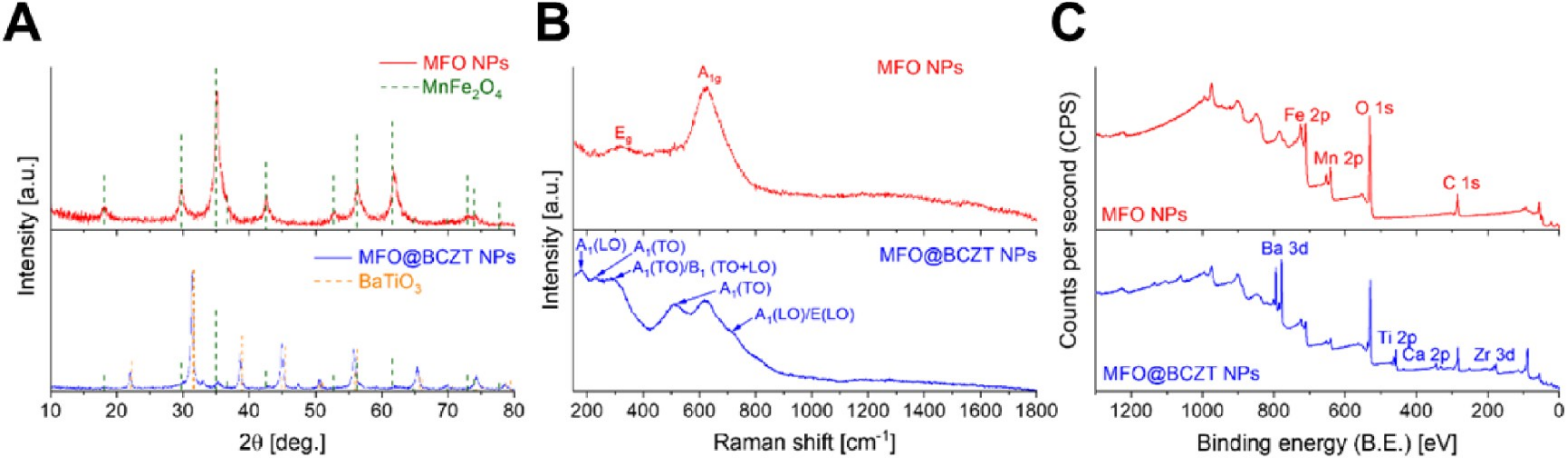
Analysis of the crystalline structure of MFO NPs and MFO@BCZT MENPs. (A) XRD patterns of MFO NPs (red), BT (blue), and MFO@BCZT MENPs (black). (B) Raman spectra of MFO@BCZT MENPs (top) and MFO NPs (bottom) using a 528 nm excitation wavelength and a maximum power of 100 mW. All characteristic peaks of BCZT, identified in blue, confirm the formation of ferroelectric BCZT phases on the surface of MFO NPs.

**Table 1:** Relative Phase Composition of the Synthesized Core MFO NPs and Core–Shell MENPs

NPs	Phase	COD card number	Content, %
MFO	spinel MnFe_2_O_4_	#10-0319	100
MFO@BCZT	perovskite BaTiO_3_	#75-0279	61
	spinel MnFe_2_O_4_	#10-0319	39

Specific chemical states on the surface of the NPs were determined using high-resolution XPS spectra (Figure S1). The deconvolution of the C 1s region for MFO cores and core–shell MFO@BCZT MENPs yielded three typical peaks of functional groups C–C/C–H (285 eV), C–O (286.5 eV), and O–C=O (288.7 eV), corresponding to adventitious carbon and citric acid.^52,53^ Furthermore, for MFO cores, a small peak was detected at 283 eV that can be associated with carbide owing to the partial decomposition of citric acid.^52,54^ Fitting of the O 1s region uncovered a high-intensity peak at 530 eV matching lattice oxygen (metal oxide) from the structure of MFO and BCZT for cores and core–shell NPs, as well as confirmed functional groups C–O at 532 eV and O–C=O at 533.3 eV, which may be attributed to the presence of citric acid used for surface functionalization.^52,53,55^ On the other hand, the observed changes in the intensity after BCZT shell formation can be explained by oxygen vacancies and hydroxyl groups on the surface of NPs.^55^ Moreover, because the BCZT shell formation was performed in a concentrated NaOH solution, a small Auger peak of Na was observed in the O 1s region of core–shell MFO@BCZT MENPs. The fitting of the Mn 2p spectra revealed an intense peak of Mn^2+^ and a less intense peak of Mn^3+^, indicating partial oxidation of Mn^2+^ to Mn^3+^ during the synthesis of MFO cores. These Mn^3+^ ions can partially occupy tetrahedral (denoted as Th) interstitial sites, which was noted in the Fe 2p region together with octahedral (denoted as Oh) interstitial sites matching the cubic spinel-type structure.^56^ It is worth mentioning that the profile of Mn 2p and Fe 2p regions for MFO@BCZT MENPs stayed clear-cut but became noisier as compared to MFO cores, indicating the emergence of a thin BCZT shell, since the depth of XPS sensitivity for a solid state is approximately 3–4 nm.^57^ While the Mn 2p and Fe 2p regions are associated with MFO, other chemical regions corresponding to BCZT (Ba 3d, Ca 2p, Zr 3d, and Ti 2p) showed clearly spaced spin–orbit components. The main peaks of these regions have typical binding energies of their oxide lattice in BCZT, such as Ba 3d_5/2_ at 779.5 eV, Ca 2p_3/2_ at 346.1, Zr 3d_5/2_ at 181.6, and Ti 2p_3/2_ at 458.3 eV, confirming the formation of the BCZT compound.^52,58,59^ It is noteworthy that the reported asymmetric peak of Ba 3d_5/2_ indicates the formation of barium carbonate on the surface of perovskite BT subjected to ambient conditions owing to the oxidation of hydroxyl groups on the surface, which leads to the formation of carbonates according to the reaction Ba­(OH)_2_ + CO_2_ → BaCO_3_ + H_2_O.^60^ In the present study, a sharp symmetric peak of Ba 3d_5/2_ was documented for the core–shell MFO@BCZT MENPs. This can be ascribed to a small amount below sensitivity, resulting in an overlap with the oxide lattice of BCZT or by the complete absence of BaCO_3_ on the surface of core–shell NPs owing to the *in situ* surface functionalization with citric acid, which can limit and prevent BaCO_3_ formation.

Magnetic properties of MFO cores and MFO@BCZT MENPs are presented in Figure 3A and Table 2. As compared to polycrystalline MFO (80 emu g^–1^) and previously reported MFO NPs (61–67 emu g^–1^) with a larger size (∼77 ± 14 nm), our MFO cores (∼23 nm) manifested reduced magnetization due to the smaller particle size, in agreement with the results of HRTEM (Figure 1A).^31,61,62^ The emergence of the BCZT shell reduced the magnetization of the MFO cores from 41.4 ± 1.2 to 5.7 ± 0.2 emu g^–1^. By contrast, coercivity increased from 43 ± 1 to 69 ± 5 Oe owing to the BCZT shell formation. These changes in the magnetization and coercivity of MENPs can be explained by a tight coupling (between the MFO core and BCZT shell), which enhances the resistance force that affects the displacement of domain walls.^11,63^

**Figure 3:**
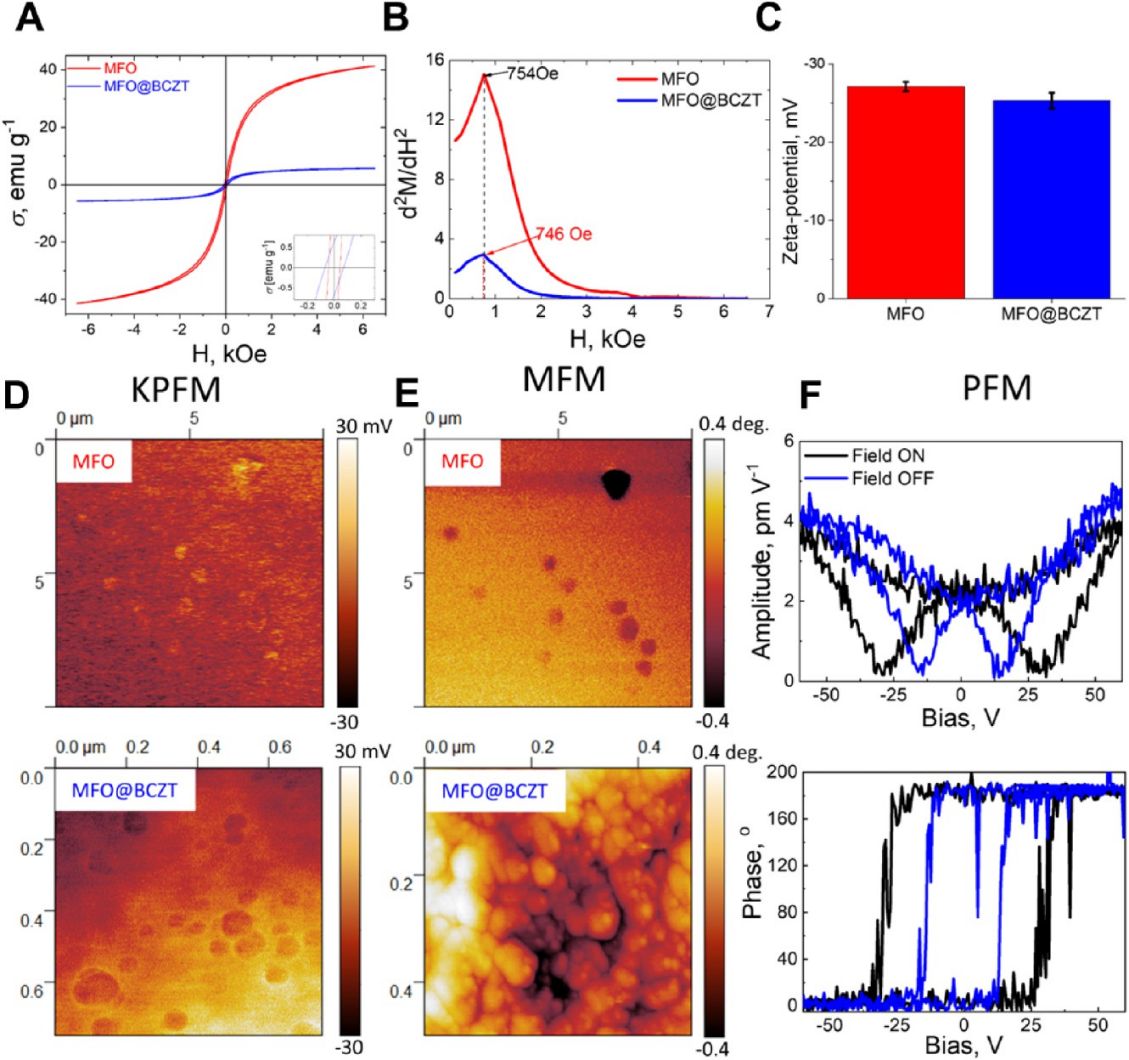
Characterization of the magnetic and electrical properties of MFO NPs and MFO@BCZT MENPs. (A) Hysteresis loops of MFO NPs (red) and MFO@BCZT MENPs (blue) recorded at 300 K with a magnetic field varying from 0 to 6.5 kOe. (B) Second-order derivative of the magnetization d^2^ *M*/d*H* ^2^. (C) ζ-potential values. (D) Representative KPFM and (E) MFM images for the studied NPs. Scale bars in the KPFM and MFM images are 500 nm. (F) The local piezoresponse (the amplitude–voltage and phase–voltage hysteresis loops) of core–shell MFO@BCZT MENPs during ferroelectric and ME characterization by PFM without or with a magnetic field (500 Oe).

**Table 2:** Magnetic Properties of MFO NPs and Core–Shell MFO@BCZT MENPs

NPs	σ_s_, emu g^–1^	σ_r_, emu g^–1^	*H*_c_, Oe
MFO	41.4 ± 1.2	2.3 ± 0.2	43 ± 3
MFO@BCZT	5.7 ± 0.2	0.7 ± 0.1	69 ± 5

*In situ* synthesis allowed us to obtain magnetic cores and MENPs with a zeta potential (ζ-potential) of −27.1 ± 0.6 and −25.3 ± 1.0 mV (Figure 3C), respectively. According to the literature, these values of ζ-potential are typical for CA-functionalized magnetic NPs at pH 7.0.^64^ Meanwhile, the absence of the difference in ζ-potential implies similar numbers of polar functional groups from CA on the surface of cores and core–shell MENPs, as confirmed by the XPS spectra (Figure S1). Figure 3D and E represents the results of Kelvin probe force microscopy (KPFM) and magnetic force microscopy (MFM) characterization of the MFO NPs and MFO@BCZT MENPs, respectively. It is evident that the average surface potential of MFO core NPs and core–shell MFO@BCZT MENPs is negative (Figure 3D), confirming the results of dynamic light scattering (DLS) (Figure 3C). This result is in good agreement with XPS data (Figures 2C and S1), which revealed polar functional groups on the surface of the NPs. The detected deflection of a nonvibrating cantilever owing to the magnetic interaction between the tip and samples during MFM measurements is demonstrated as a low dark contrast in Figure 3E, consistently with a coherent rotation of the spins both in the MFO cores and core–shell MFO@BCZT MENPs under the influence of the tip stray field. This finding indicates relatively low net anisotropy energy arising from the small size of both types of NPs (cores and core–shell MENPs) and consequently their predominant superparamagnetic behavior. These interactions between the magnetic tip and the samples are expected to occur in either superparamagnetic NPs or very soft magnetic nanostructures.^65,66^ It is worth mentioning that the MFM contrast was rather high for the MFO core as compared to MFO@BCZT MENPs. The epitaxial nonmagnetic BCZT shell can result in weakening the interparticle dipole–dipole interaction and canting the surface magnetic spins of the core, causing the overall magnetic moment to deviate from alignment.^67^ It is still challenging to determine the preferred magnetization direction for superparamagnetic or very soft magnetic materials using MFM because their magnetization can randomly flip or change rapidly under the presence of the tip’s magnetic field and external fields.^68,69^

Finally, the ferroelectric and ME behavior of single core–shell MFO@BCZT MENPs was evaluated by piezoresponse force microscopy (PFM) measurements with a direct current bias voltage and an external in-plane SMF applied to switch the direction of polarization and characterize the ME coupling, respectively. The corresponding piezoresponse (amplitude vs bias voltage) and phase hysteresis loops are displayed in Figure 3F. It can be stated that the phase curves are switched at the angles of 0° and 180° with and without the external magnetic field, indicating a ferroelectric nature rather than an electrostatic response. As-synthesized core–shell MFO@BCZT MENPs showed an effective local in-plane piezoresponse up to 5 pm/V. At the same time, the coercive voltage of the MENPs featured a large shift when MENPs were subjected to the external magnetic field, thus implying an effective transfer of the generated strain in the magnetostrictive MFO core to the BCZT shell, thereby causing polarization reversal in BCZT. The shift of positive coercive voltage (12.67 V) was greater than that of the negative ones (12.06 V). The direct ME (α_ME_) coefficient of the core–shell MFO@BCZT MENPs, which reflects the coupling between piezoelectric and magnetostrictive phases, was then calculated using eq 1 and was found to be α_ME_ = (12.67 – 12.06 V)/(2 × 4 nm)/500 Oe = 152 × 10^4^ mV cm^–1^ Oe^–1^. This value is within the range of the most recently reported ME coupling values for core–shell MENPs.^11,70–72^ Remarkably, although CoFe_2_O_4_-based systems exhibit the highest ME response reported to date (1.40 × 10^6^ mV cm^–1^ Oe^–1^), concerns regarding cobalt-related toxicity limit their use in biomedical applications.^73^ In contrast, we successfully engineered core–shell MENPs with an ultrathin (∼4 nm) crystalline heteroepitaxial BCZT shell on the surface of the MFO core. This design achieved a comparable ME response (152 × 10^4^ mV cm^–1^ Oe^–1^) while potentially mitigating biocompatibility concerns due to the favorable biological profiles of manganese and BCZT.^28,32,74–76^ Moreover, BCZT is reported to exhibit a significantly stronger piezoelectric response than that of BT, further supporting its potential for biomedical applications.^77^ It is worth noting that the ME response of the developed MFO@BCZT MENPs may surpass both the calculated value and the above-mentioned value for Co-based MENPs in the literature. This enhancement is attributed to the presence of an ultrathin nanocrystalline BCZT shell, thinner than those typically considered in average ME response estimations (Figure 1A). Collectively, these findings position the MFO@BCZT MENPs as highly promising candidates for bone repair therapies.

### Evaluation of Behavior of MFO NPs and MFO@BCZT MENPs in Culture Medium

Before assessing the internalization of MFO NPs and MFO@BCZT MENPs by the cells, we first evaluated their behavior in culture media, as their dynamics are influenced by gravitational sedimentation forces, Brownian motion, and interparticle interactions.^78^ Additionally, factors such as NP size, shape, and surface charge, along with medium properties such as viscosity, temperature, and volume, can cause them to settle, diffuse, or aggregate, ultimately impacting their cellular uptake.^78–80^

To simulate *in vitro* assays, we dispersed the NPs at various concentrations in the culture medium and incubated the suspensions overnight at 37 °C. After incubation, the absorbance of the supernatant was measured and compared to the absorbance values obtained following NP resuspension.^79^ The results revealed that both types of NPs sedimented regardless of the concentration tested, as evidenced by the significant differences between the absorbance values before and after resuspension (Figure 4Ai and ii). While sedimentation can increase the NP concentration near the cells and potentially enhance internalization, it may also lead to high intracellular doses of NPs. This high dosage has been identified as a critical factor in iron-mediated toxicity.^80,81^ This occurs when iron metabolism pathways are overwhelmed, leading to the release of free iron ions that react with hydrogen peroxide, generating highly toxic hydroxyl radicals that cause cellular damage.^81^ Still, the threshold for toxicity remains controversial and depends on several factors, including core composition, surface coating, particle size, shape, cell type, donor variability, and cellular status.^81,82^ Therefore, evaluating NP biocompatibility at each tested concentration is essential and will be addressed in subsequent sections.

**Figure 4:**
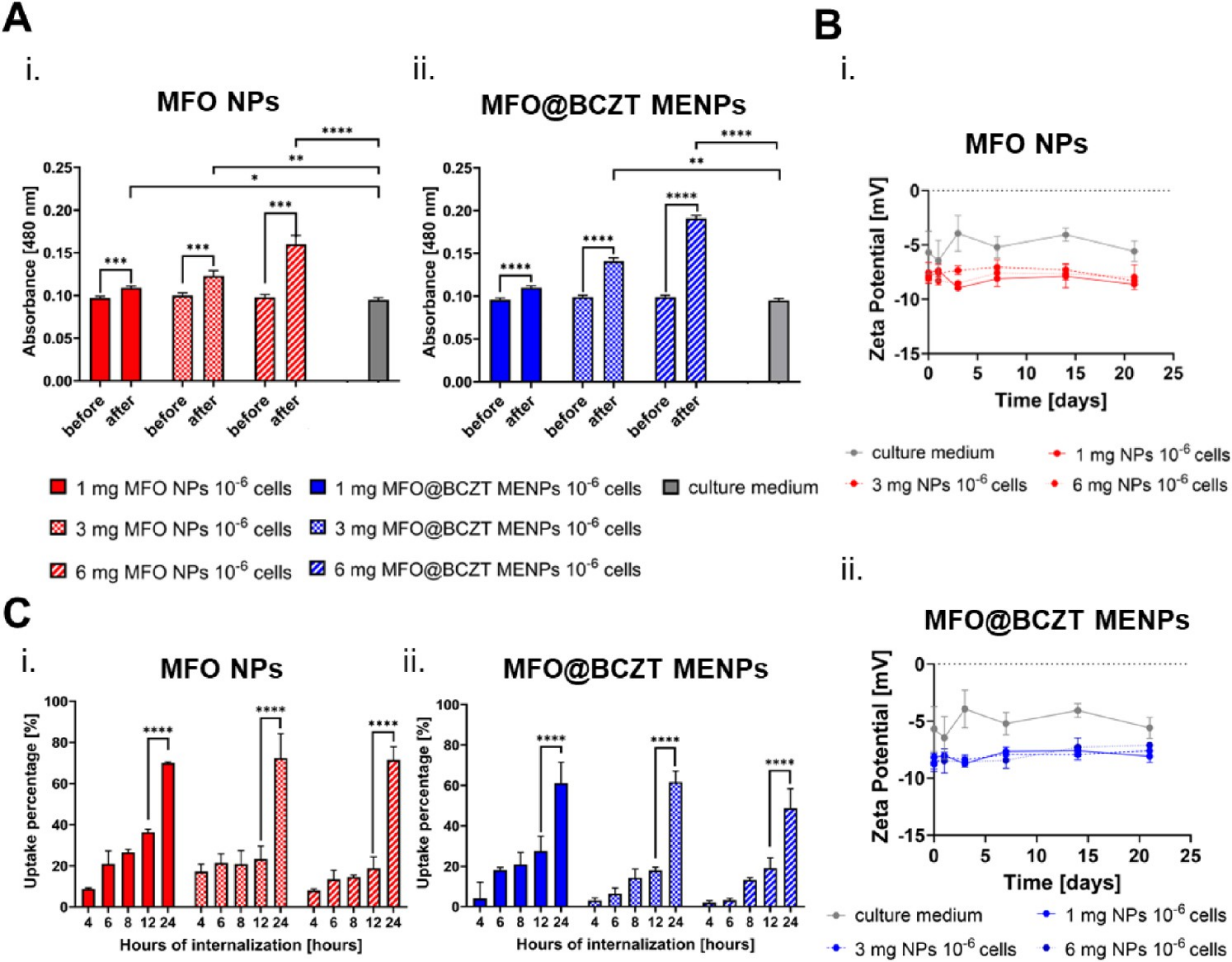
Stability analysis of MFO NPs and MFO@BCZT MENPs and their uptake by hASCs. (A) Assessment of the stability of (i) MFO NPs and (ii) MFO@BCZT MENPs in the culture medium based on absorbance measurements of the supernatant before and after NPs resuspension, following an overnight incubation at 37 °C. Culture medium without NPs served as the control. Data in (i) and (ii) are presented as mean ± standard deviation (SD) (*n* = 3). (B) Surface charge characterization of (i) MFO NPs and (ii) MFO@BCZT MENPs in the culture medium, assessed by monitoring the ζ potential over a 21-day period under conditions simulating the *in vitro* experiments. Data in (i) and (ii) are presented as mean ± SD (*n* = 3). (C) Analysis of the (i) MFO NPs and (ii) MFO@BCZT MENPs uptake by hASCs by varying the NP concentration seeded with cells and the internalization time up to 24 h. Data in (i) and (ii) are presented as mean ± SD (*n* = 3).

Another critical parameter is the NP surface charge, which influences both interparticle interactions and NP–cell membrane interactions in biological media.^83^ To assess this, we monitored changes in the ζ-potential of both types of NPs dispersed in the culture medium over 21 days. The suspensions were maintained at 37 °C under sterile conditions, with medium exchanges performed every 3 days via magnetic actuation to faithfully recreate the *in vitro* assay conditions and prevent media acidification. Figure 4Bi and ii show that ζ-potential values remained relatively stable over time, indicating that either MFO NPs or MFO@BCZT MENPs maintained a consistent surface charge in the biological medium, regardless of the concentration tested. The average ζ-potential values for both core and core–shell NPs were below −10 mV, a threshold associated with increased flocculation risk.^83,84^ This observation aligns with the sedimentation behavior, as flocculation promotes aggregation and enhances sedimentation through gravitational forces.^85^ It is important to note that the ζ-potential values presented here differ from those in Figure 3C due to the formation of a protein corona. Since the culture medium was supplemented with fetal bovine serum (FBS), the presence of positively charged proteins likely altered the NP surface chemistry by coating the originally grafted surface groups, thereby reducing the net negative charge of the NPs.^86^ Nonetheless, considering that cell membranes are rich in negatively charged components, this reduction in NP surface negativity may favor endocytosis by improving electrostatic attraction to the cell membrane.^87^

### Analysis of Internalization of MFO NPs and MFO@BCZT MENPs by hASCs

As this study aims to compare the ability of magnetic core MFO NPs and core–shell MFO@BCZT MENPs to promote osteogenic differentiation in hASCs, we first confirmed the stemness of the hASCs prior to their magnetization with NPs. Flow cytometry analysis revealed that the hASCs expressed negative endothelial markers, while over 98% expressed positive mesenchymal stem cell markers, consistent with their stem cell profile (Figure S2).

Following this validation, we investigated the uptake of both core and core–shell NPs by the hASCs. For this purpose, hASCs were incubated with varying concentrations of NPs (1, 3, and 6 mg of NPs per million of cells) for different internalization periods (6, 8, 12, and 24 h) in the absence of a magnetic field. The uptake of the NPs was quantified by measuring the absorbance in both the culture medium and cell lysate, following membrane permeabilization with Triton X-100 and mechanical scraping to release intracellular NPs. The percentage of internalized NPs was calculated as the ratio of the absorbance measured within cells to the total absorbance (cells + culture medium).

Results revealed that the percentage of the cellular uptake increased over time, with the highest uptake occurring after 24 h of internalization, regardless of the NPs’ type and concentration used (Figure 4Ci and ii). Specifically, when comparing the 12-hour mark with the 24-hour mark , MFO NPs showed a 1.9-fold increase in absorbance (****p < 0.0001) at a concentration of 1 mg per 10^6^ cells, a 3.1-fold increase (***p < 0.0001) at a concentration of 3 mg per 10^6^ cells, and a 3.9-fold increase (***p < 0.0001) at a concentration of 6 mg per 10^6^ cells. Similarly, MFO@BCZT MENPs showed 2.2-fold, 3.4-fold, and 2.5-fold increases in absorbance (****p < 0.0001) at concentrations of 1, 3, and 6 mg per 10^6^ cells, respectively. These differences in NP uptake may be attributed to variations in their chemistry and size, both of which are known to influence cellular internalization.^88–90^ While extending the internalization period could enhance the intracellular uptake of NPs, it would also alter the NP-to-cell ratio due to ongoing cell proliferation. Given that hASCs at the passage used have a reported doubling time of 30 to 35 h, we limited the maximum internalization time to 24 h to preserve an accurate NP-to-cell ratio.^91^

As no magnetic field was used during the uptake process of the NPs by hASCs, we excluded electroporation as a possible entry mechanism. We therefore hypothesized that the NPs were internalized by hASCs through conventional endocytic pathways, such as clathrin-/caveolin-mediated endocytosis, clathrin-/caveolin-independent endocytosis, phagocytosis, and/or micropinocytosis. As NPs are typically enclosed within endosomes and transported to lysosomes after internalization via any route, we performed LysoTracker probe staining, which labels lysosomes in red (Figure S3).^89,92^ This staining revealed higher lysosome expression under conditions where NPs were present, with no significant differences observed as their concentration increased. These observations align with the data shown in Figure 4Ci and ii.

### Assessment of the Biological Performance of MFO NPs and MFO@BCZT MENPs

To determine whether the MFO NPs and MFO@BCZT MENPs were biocompatible or cytotoxic, live/dead assays were performed after 1, 3, and 7 days of culture, following NPs’ internalization by hASCs. Compared to the control group, which consisted of cells not exposed to NPs, both MFO NPs and MFO@BCZT MENPs demonstrated similar behavior, supporting cell proliferation over the 7 days of culture with minimal cell death (Figures 5A and 6A).

**Figure 5:**
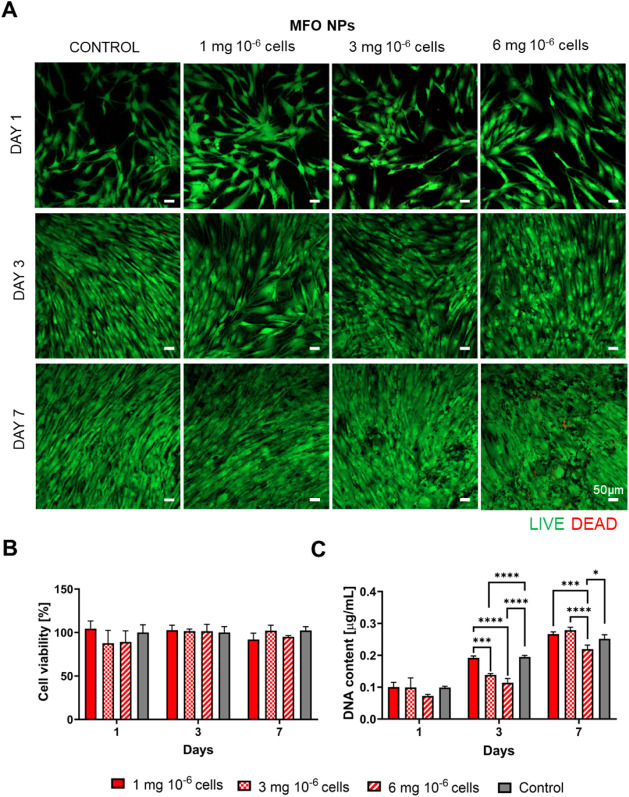
Viability characterization of hASCs exposed to various concentrations of MFO NPs. (A) Representative live/dead images of hASCs treated with MFO NPs after 1, 3, and 7 days in culture. Nonmagnetized cells were used as a control. Scale bar: 50 μm. Images were acquired using a confocal microscope coupled with a 10× objective. (B) Cell metabolic activity assessed by the MTS colorimetric assay. (C) Cell proliferation measured by DNA quantification. Data in B and C are presented as mean ± SD (*n* = 4).

**Figure 6:**
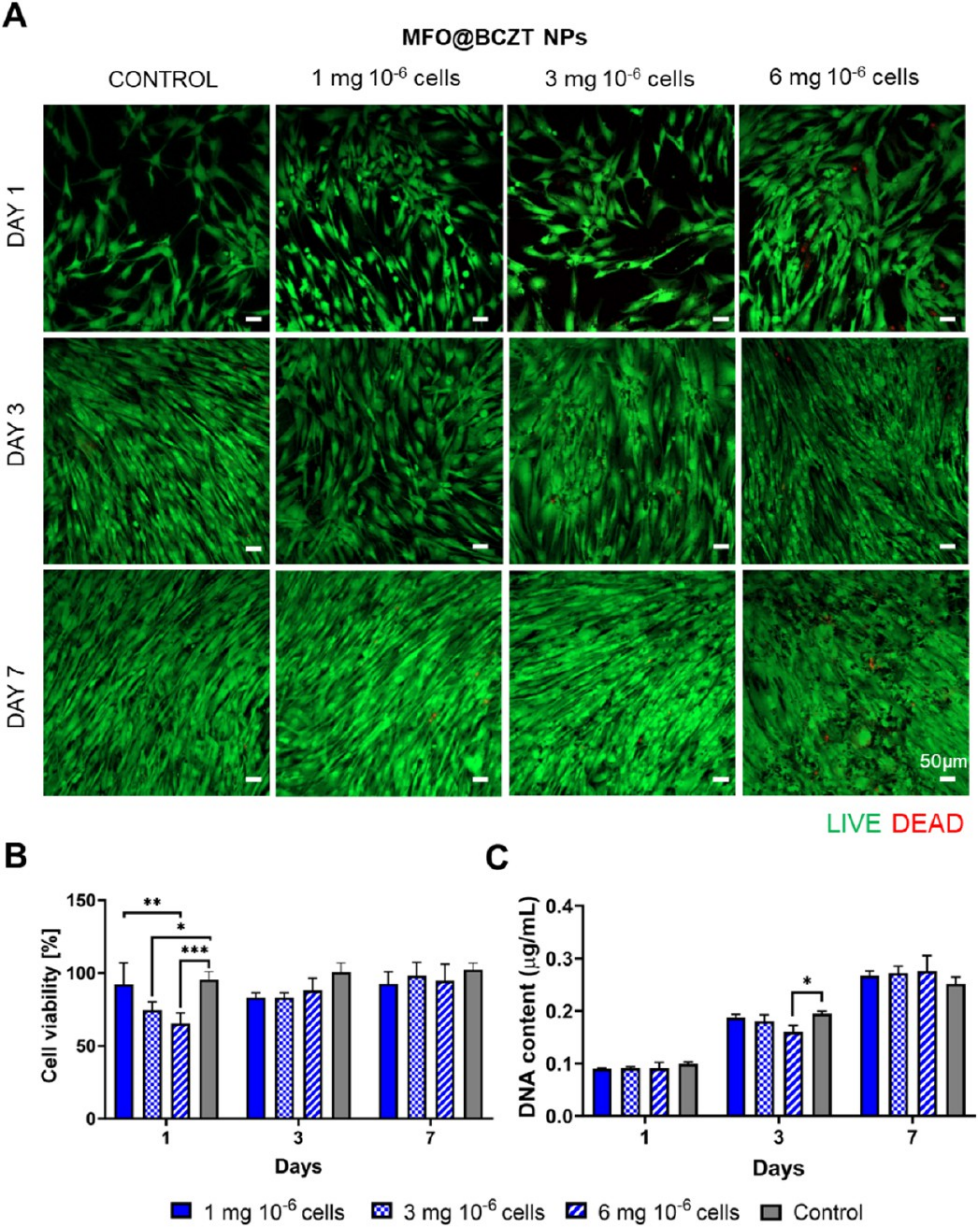
Viability characterization of hASCs exposed to various concentrations of MFO@BCZT MENPs. (A) Representative live/dead images of hASCs treated with MFO@BCZT MENPs after 1, 3, and 7 days in culture following NP internalization. Nonmagnetized cells were used as a control. Scale bar: 50 μm. Images were acquired using a confocal microscope with a 10× objective. (B) Cell metabolic activity assessed by MTS colorimetric assay. (C) Cell proliferation measured by DNA quantification. Data in B and C are presented as mean ± SD (*n* = 4).

Similarly, cell morphology analysis showed that the organization of F-actin microfilaments was not affected by NP internalization, as evidenced by increased cell elongation over time and the formation of a densely interconnected network of elongated cells by day 7 (Figure S4A and B). To further assess the effect of the different concentrations of NPs on cell viability, we measured cell metabolic activity and performed DNA quantification. As illustrated in Figures 5B and 6B, a slight decrease in viability was observed on day 1 across most concentrations for MFO NPs and MFO@BCZT MENPs, with a significant reduction noted for MFO@BCZT MENPs. Nonetheless, for both types of NPs, an exception was observed at a concentration of 1 mg of NPs per 10^6^ cells, where viability matched that of the control. For the remaining days, no significant differences in viability were detected, and cells remained viable throughout the entire culturing period. Regarding the DNA quantification (Figures 5C and 6C), results indicated an increase in DNA content over time, suggesting that the cells were proliferating, in agreement with the live/dead findings. As for both MFO and MFO@BCZT conditions, the formulation containing 1 mg of NPs per 10^6^ cells showed the lowest variation in terms of metabolic activity and DNA content compared to the control group, while still conferring cells a magnetic responsiveness; this concentration was chosen for further experiments.

### Assessment of Magnetic Field Stimulation on the Viability and Metabolic Activity of hASC Spheroids Containing MFO NPs or MFO@BCZT MENPs

Building on the demonstrated biocompatibility of the core (MFO) and core–shell (MFO@BCZT) NPs and the ability of magnetic stimulation to promote the assembly of cells into complex architectures, we next investigated the formation and behavior of hASC spheroids containing these NPs. The use of spheroids as a 3D culture model was motivated by their architectural and organizational resemblance to native tissues, particularly in terms of cell–cell and cell–extracellular matrix contacts.^90,93^ These features improve physiological relevance and provide a more predictive platform for assessing the effects of magnetic stimulation on spheroid viability, proliferation, and differentiation.^93^ For this purpose, previously magnetized hASCs were seeded on ultralow adhesion well plates, centrifuged, and incubated for 72 h with a magnet beneath each well to promote spheroid aggregation (Figure 7Ai). To better replicate the *in vivo* conditions, magnetized spheroids were subsequently encapsulated in gelatin methacryloyl hydrogels (GelMA) to emulate the natural embedding of tissue within the extracellular matrix environment.

**Figure 7:**
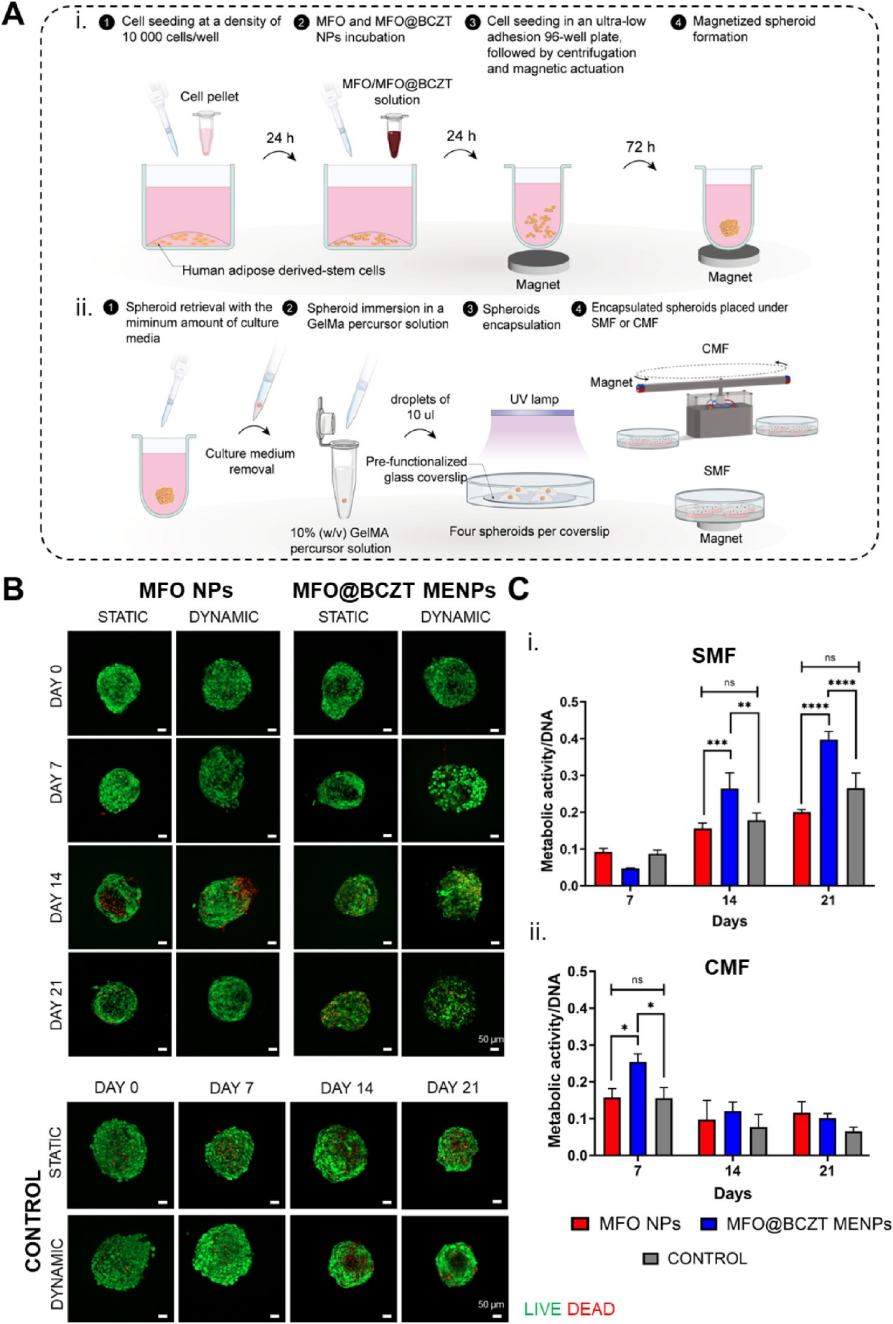
Viability and proliferation of spheroids containing MFO NPs and MFO@BCZT MENPs under SMF and CMF stimulations. (A) Schematic illustration of the strategy followed to produce magnetized spheroids. Image created with Adobe Illustrator. (B) Representative live/dead images captured via confocal laser microscopy under a 10× objective at 0, 7, 14, and 21 days in culture. Nonmagnetized spheroids (control condition) served as controls and underwent the same preparation method, with the exception that the 3-day incubation for spheroid formation was conducted without magnetic stimulation. Scale bar: 50 μm. (C) Assessment of cell metabolic activity in magnetized and nonmagnetized spheroids after (i) SMF and (ii) CMF exposure over a 21-day period. Results were normalized to DNA content. Data are presented as mean ± SD (*n* = 3).

To prevent any unintended movement of the magnetized tissues during magnetic stimulation, hydrogels containing the spheroids were immobilized on glass coverslips through a surface functionalization process. This setup ensured that magnetic fields were the only mechanical stimuli applied. For quantitative analysis, four hydrogels (each embedding a single spheroid) were fixed per coverslip and subsequently cultured either under SMF or CMF, as illustrated in Figure 7Aii.

The results indicated that both groups of magnetized spheroids remained viable up to 21 days under SMF or CMF exposure (Figure 7B). Neither the MFO NPs nor the BCZT piezoelectric shell in MFO@BCZT MENPs exhibited cytotoxic effects on cells compared to nonmagnetized spheroids under the same magnetic field conditions, supporting previous reports on the biocompatibility of these materials.^28,32,33,77,94^ Although dead cells were observed in all groups, particularly from day 14 onward, these occurrences were attributed to the high degree of cellular compaction inherent to spheroids, which restricts nutrient and oxygen diffusion toward the core.^95^

To assess the influence of MFO NPs and MFO@BCZT MENPs on spheroid proliferation under magnetic stimulation, we analyzed their metabolic activity normalized to the DNA content. Under SMF conditions, cellular metabolic activity gradually increased in all groups (Figure 7Ci). Notably, spheroids with MFO@BCZT MENPs exhibited significantly higher metabolic activity at days 14 and 21 than those with MFO NPs or nonmagnetized controls, which showed relatively stable activity throughout the culture period. These observations suggest that the piezoelectric shell significantly enhances cellular metabolism, consistent with earlier reports.^7^ It is likely that SMF induces mechanical stress in the magnetostrictive MFO core, which is then transmitted to the BCZT piezoelectric shell, generating localized electrical signals. These electrical cues are known to promote biological processes such as cell proliferation, migration, and differentiation, supporting the basis of electrical stimulation therapies.^3,7,9^ Under CMF, similar trends were observed at day 7, reinforcing the importance of the piezoelectric shell in enhancing cellular metabolism, especially during energy-demanding processes such as tissue regeneration and repair (Figure 7Cii).^9^ However, by days 14 and 21, the metabolic activity had plateaued across all groups, showing no significant differences.

### Evaluation of Magnetic Stimulation on the Osteogenic Differentiation of Spheroids Incorporating MFO NPs or MFO@BCZT MENPs

Given the established role of magnetic fields in promoting the osteogenic differentiation of hASCs, we then analyzed the expression of key bone-specific markers in the osteoblast lineage.^36,96–98^ Bone formation is typically divided into three main stages: cell proliferation, extracellular matrix maturation, and matrix mineralization.^7,99,100^ Each stage is regulated by the expression of specific osteoblast markers, as illustrated in Figure 8A.^100,101^ During the proliferation stage, extracellular proteins such as collagen type I (COL I), alkaline phosphatase (ALP), and fibronectin are expressed along with bone-related genes such as *Runx2* and *Osterix*. The maturation phase is marked by sustained expression of ALP and COL I, along with increasing levels of osteopontin, osteocalcin, and bone sialoprotein. In the mineralization stage, osteopontin and bone sialoprotein are dominant, while ALP, COL I, and osteocalcin expression decline. At the end of this process, fully matured osteoblasts may become embedded within the mineralized matrix as osteocytes, transition into inactive quiescent bone-lining cells, or undergo apoptosis.^99,100^

**Figure 8:**
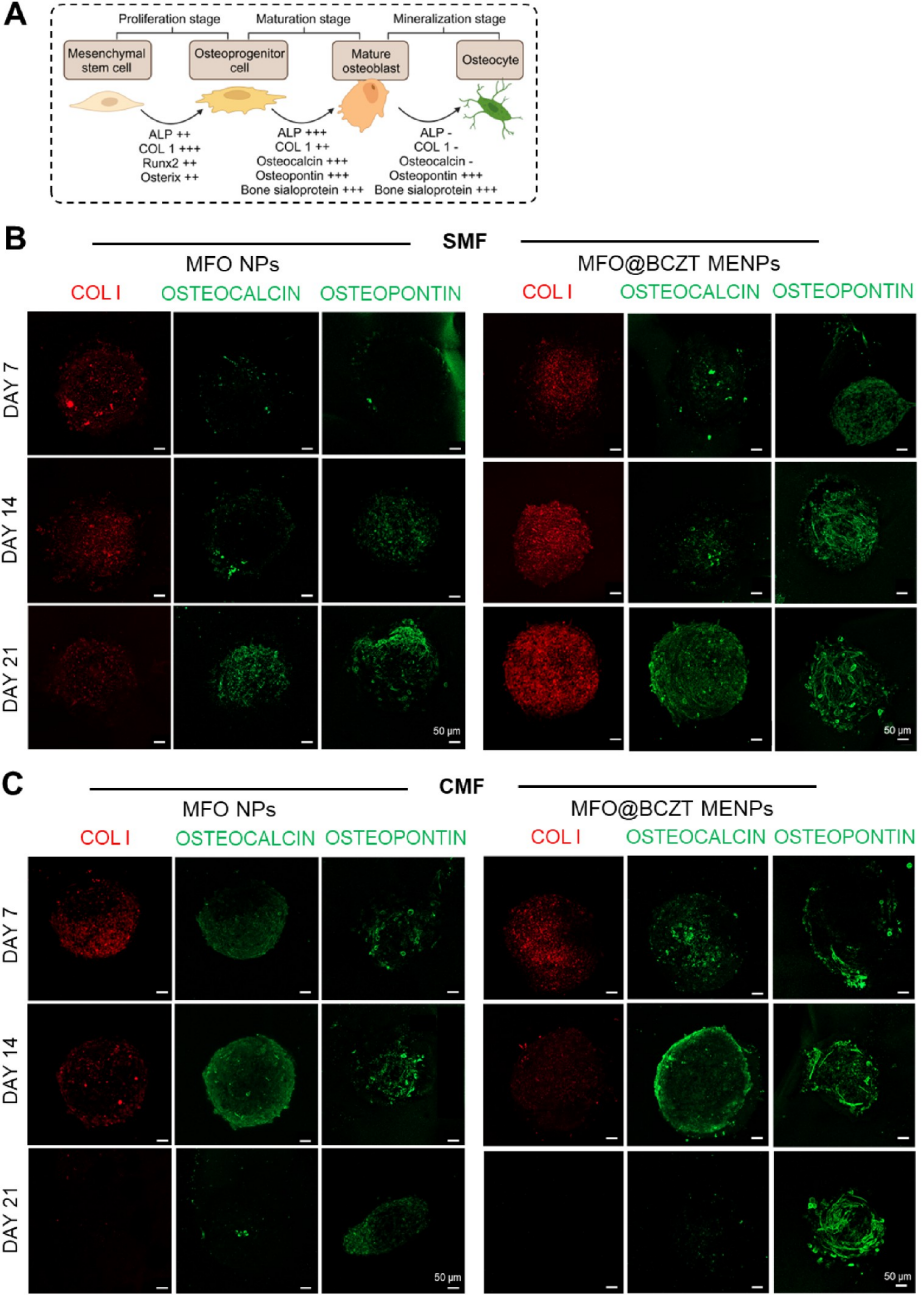
Osteogenic evaluation of hASC spheroids incorporating MFO NPs or MFO@BCZT MENPs cultured under SMF or CMF stimulation. (A) Schematic illustration of key osteogenic markers expressed at each stage of osteogenic differentiation. Image created with Biorender.com and adapted from elsewhere.^100^ (B) Representative confocal microscopy images showing COL I (red), osteocalcin (green), and osteopontin (green) expression after 21 days under SMF or (C) CMF culture conditions. Images were acquired under a 10× objective. Scale bar: 50 μm.

Based on these events, osteogenic markers are typically categorized into early- and late-stage markers. Early markers include ALP, COL I, *Runx2*, and *Osterix*, while late markers include osteocalcin, osteopontin, and bone sialoprotein.^7,100^ Therefore, to compare whether magnetic MFO NPs and MFO@BCZT MENPs could induce the osteogenic differentiation of hASCs, we evaluated the expression of COL I, osteocalcin, and osteopontin in magnetized spheroids via immunofluorescence staining following exposure to SMF and CMF conditions (Figure 8B and C). For clarity of visualization, these figures display only the individual fluorescence channels corresponding to each marker. The same images with the cell nuclei stained by DAPI are provided in Figure S5A and B.

The results revealed that under SMF conditions, both MFO and MFO@BCZT-treated spheroids showed increased expression of COL I, osteocalcin, and osteopontin over the 21 days in culture (Figures 8B and S5A). This increase was clearly visible on days 14 and 21 for the MFO@BCZT group compared to that of the MFO, as evidenced by the higher fluorescence intensity and larger stained area. Interestingly, these observations were consistent with the enhanced metabolic activity in the MFO@BCZT group at the same time points (Figure 7Ci). As previously reported, this enhancement appears to be associated with increased deposition of COL I and matrix mineralization.^102^ In fact, COL I deposition is one of the earliest indicators of mesenchymal stem cell commitment to the osteogenic lineage, providing the framework necessary for mineralization.^103^ Osteocalcin and osteopontin, in turn, are deposited in later stages of differentiation and play important roles in bone matrix organization and mineral deposition by coordinating both cell-matrix and cell-mineral interactions.^104^ Therefore, the upregulated expression of these three markers may suggest that spheroids treated with MFO@BCZT MENPs have progressed to a more advanced stage of matrix maturation compared to those treated with core MFO NPs (see Figure 8A for the timeline expression of the osteogenic markers). This conclusion is further corroborated by differences in the staining patterns of osteocalcin and osteopontin, which indicated improved bone extracellular matrix organization and deposition in the MFO@BCZT group. By day 21, osteocalcin showed a predominantly filamentous distribution in spheroids treated with MFO@BCZT MENPs. In contrast, those treated with MFO NPs showed a more clustered distribution, suggesting a less mature extracellular matrix organization (Figures 8B and S5A; see osteocalcin staining). These differences were even more pronounced for osteopontin staining. In the MFO@BCZT group, filamentous-like structures were clearly visible as early as day 14, whereas in the MFO group, such organization was only observed by day 21 and covered a smaller area (Figures 8B and S5A, osteopontin staining). Since the only distinguishing factor between the two types of NPs is the presence of the piezoelectric shell, we attributed the enhanced differentiation observed in the MFO@BCZT group to the activation of the piezoelectric effect under magnetic stimulation. This generates synergistic electrical and magnetic cues that accelerate osteogenic differentiation.

Under CMF conditions, although both groups of NPs exhibited similar patterns in bone marker expression, their individual expressions did not follow the trends observed under SMF conditions (Figures 8B,C, S5A and B). COL I expression reached its maximal expression on day 7, decreased on day 14, and became undetectable by day 21 (Figures 8C and S5B). Osteocalcin expression appeared to increase up to day 14 but decreased thereafter, becoming barely detectable by day 21. In contrast, osteopontin demonstrated a gradual increase throughout 21 days of culture. The timeline illustrated in Figure 8A suggests that spheroids treated with CMF had progressed to the state of mineralization, indicating that osteogenic differentiation had occurred earlier under CMF compared to SMF. This hypothesis was further corroborated by the presence of filamentous-like structures as early as day 7 in both NP groups and by the earlier deposition of bone markers under CMF, which aligned with the peak in metabolic activity observed on day 7 (Figure 7Cii). Previous studies have indicated that the decline in metabolic activity observed at later time points (Figure 7Cii) may be attributed to the transition of some mature osteoblasts into apoptotic cells. This evidence is consistent with the decrease observed in metabolic activity, corroborating our hypothesis that CMF stimulation can promote a more advanced state of differentiation compared to SMF.^99,100,102^ However, due to similarities in bone marker expression observed through immunofluorescent assays, no conclusions could be drawn regarding the role of the piezoelectric shell under CMF conditions.

It is also well established in the literature that controlled magnetic field conditions alone can promote osteogenic differentiation in various nonmagnetized cell types, including hASCs.^40,105–110^ Given these facts, we analyzed whether magnetizing cells with MFO NPs or MFO@BCZT MENPs and then exposing the resulting engineered microtissues to SMF or CMF would facilitate osteogenic differentiation compared to nonmagnetized tissues exposed to the same magnetic field conditions. Figure S6 depicts the staining expression of COL I, osteocalcin, and osteopontin in nonmagnetized spheroids exposed to the same SMF and CMF conditions.

Under SMF, COL I expression became detectable at day 7 and appeared to increase over the 21 days in culture, while osteocalcin and osteopontin were detectable only on day 21. As mentioned above, this pattern aligns with the metabolic activity data in Figure 7Ci. In contrast, under CMF, both COL I and osteocalcin were visible on days 7 and 14, but declined by day 21, while osteopontin expression increased over time, further aligning with the metabolic activity data depicted in Figure 7Cii. Notably, the earlier expression of the late marker’s osteocalcin and osteopontin, in nonmagnetized spheroids cultured under CMF, reinforced the stimulatory effect of CMF in accelerating osteogenic differentiation compared to SMF, as already observed in magnetized spheroid groups.

Overall, based on the timeline of osteogenic marker expression (Figure 8A), it is anticipated that the CMF parameters applied in this study can also stimulate the osteogenic differentiation of nonmagnetized cells, while a delay in osteogenic differentiation is expected under SMF stimulation. This hypothesis is consistent with previous findings, indicating that the effect of magnetic fields on hASCs’ osteogenic differentiation depends on various factors such as frequency, field strength, and the presence or absence of osteogenic induction medium, although the latter variable was not herein studied.^40^ Nonetheless, based on staining patterns and area coverage, the combined effect of magnetic field stimulation and the presence of magnetic NPs appear to have a greater impact on osteogenic differentiation, aligning with findings from other studies.^98,111^

### Osteopontin and VEGF Cytokine Release

In addition to immunofluorescence staining of osteogenic markers, we evaluated the release profiles of osteopontin and vascular endothelial growth factor (VEGF) using enzyme-linked immunosorbent assay (ELISA) kits (Figure 9A and B). Under both SMF and CMF conditions, osteopontin expression increased over time, with the highest levels detected for magnetized spheroids treated with MFO@BCZT MENPs (Figure 9Ai and Aii). Notably, osteopontin levels were consistently higher under CMF compared with SMF, regardless of the time point. In contrast, VEGF release either decreased over time or remained low under both SMF and CMF conditions, with more pronounced downregulation observed under CMF (Figure 9Bi and Bii). According to previous studies, a downregulation in VEGF expression combined with increased expression of osteogenic markers indicates progression toward osteogenic differentiation.^35,36^ In response to mechanical forces, mesenchymal stem cells, including hASCs, are known to release VEGF, an important angiogenic factor involved in vascular network formation and in the secretion of growth factors that stimulate osteogenesis.^35,36,112^ As hASCs commit to the osteogenic lineage, their ability to promote angiogenesis diminishes, reflected by a decrease in VEGF secretion.^35,36,112–114^

**Figure 9:**
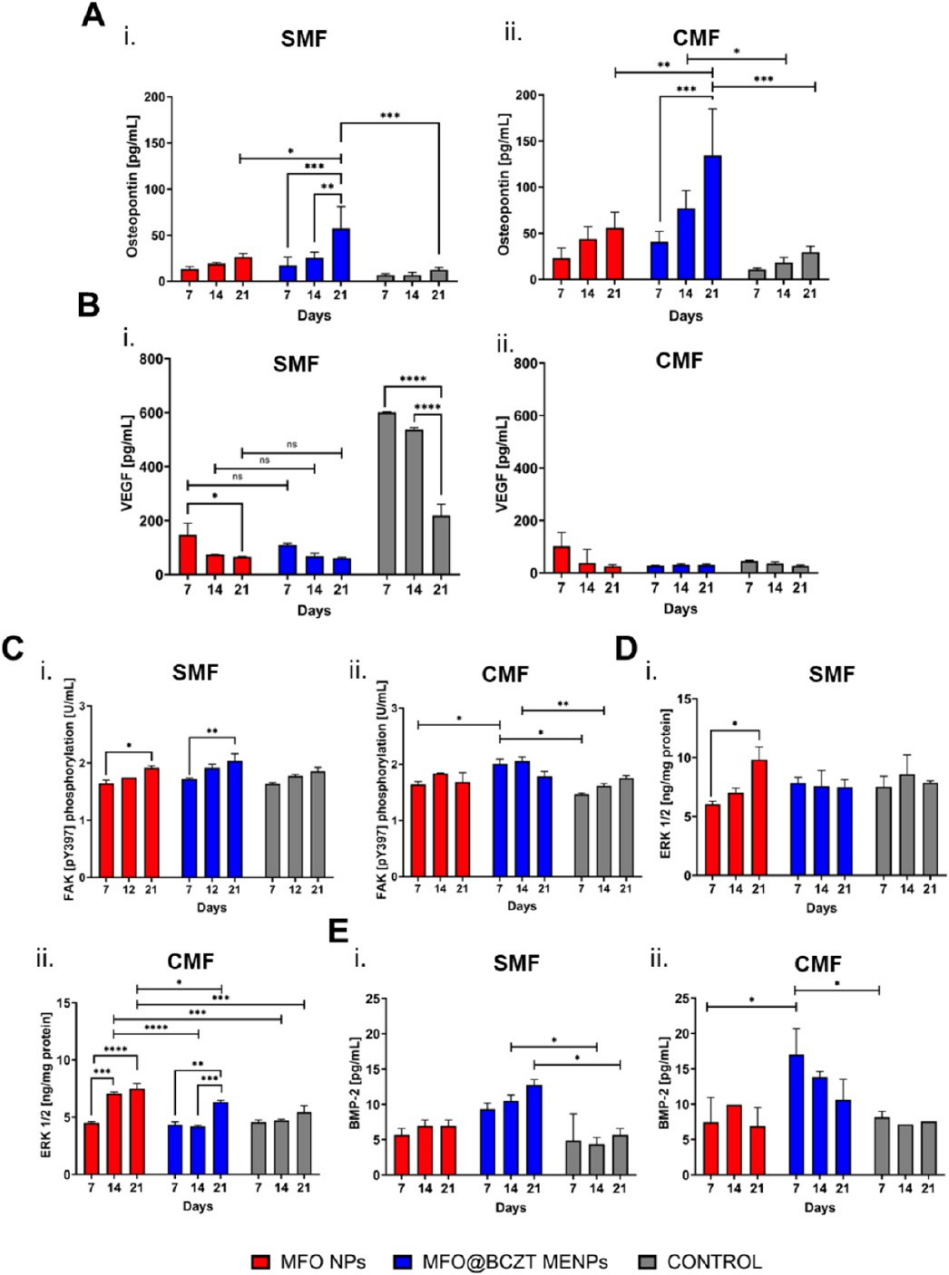
Analysis of osteopontin and VEGF release profiles and investigation of signaling pathways potentially involved in promoting osteogenic differentiation. (A and B) The quantification of osteopontin and VEGF, respectively, for cells cultured under (i) SMF and (ii) CMF conditions. (C, D, and E) The quantification of FAK [pY397], ERK 1/2, and BMP-2, respectively, for cells cultured under (i) SMF or (ii) CMF conditions. β-catenin expression was also assessed, but no expression was detected. For all paired graphs, the *y*-axis scale was kept consistent to facilitate easier comparison. Data are presented as mean ± SD (*n* = 3).

In this study, VEGF downregulation in magnetized spheroids cultured under CMF coincided with an increased expression of osteopontin from day 7 to day 21 (Figure 9Aii and Bii). Conversely, under SMF conditions, VEGF expression was initially higher on day 7 (relative to CMF) but declined over time, coinciding with a simultaneous increase in osteopontin expression from day 7 to day 21 (Figure 9Ai and Bi). These trends confirmed that osteogenic differentiation occurred in all magnetized spheroid groups regardless of the magnetic field conditions. However, the strongest downregulation of VEGF and the highest osteopontin expression under CMF support our hypothesis that CMF promotes a more advanced stage of differentiation. This effect was evident in spheroids treated with either MFO NPs or MFO@BCZT MENPs, indicating that the CMF itself is a key driver of differentiation.

Interestingly, the significantly higher levels of osteopontin by day 21 in spheroids treated with MFO@BCZT MENPs under CMF compared to those treated with MFO NPs suggest that the piezoelectric shell may further enhance cell differentiation. Although this effect was not visible by immunofluorescence analysis (Figures 8C and S5B), osteopontin quantification by ELISA and suppressed VEGF levels support this conclusion. These findings may also explain the elevated metabolic activity observed on day 7 (Figure 7Cii), possibly due to the enhanced expression of bone-related markers. Consistent with earlier metabolic and immunofluorescence data (Figures 8B, S5A, and 7Cii), the piezoelectric shell also exerted a positive effect under SMF, as evidenced by the higher expression of osteopontin on day 21 in MFO@BCZT-treated spheroids compared to those treated with MFO NPs.

In nonmagnetized spheroid groups, osteopontin expression remained low under SMF conditions after day 14, aligning with the initial increase in VEGF expression on days 7 and 14, followed by a significant decline concurrent with osteopontin expression (Figure 9Ai and Bi). Under CMF conditions, VEGF expression was already downregulated by day 7 and continued to decrease throughout the 21-day culture period, while osteopontin levels gradually increased (Figure 9Aii and Bii). Altogether, the results insinuated that osteogenic differentiation also occurred in nonmagnetized spheroids cultured under SMF and CMF conditions; however, their differentiation appeared to be delayed compared to the differentiation induced by magnetized spheroid groups. Furthermore, the lower VEGF levels and higher osteopontin expression observed under CMF conditions supported the hypothesis that CMF promotes a more advanced stage of differentiation, even in the absence of magnetized cells.

### Osteogenic Signaling Pathways Triggered in Response to MFO NPs and MFO@BCZT MENPs under Magnetic Fields

To investigate the signaling pathways involved in the osteogenic differentiation of hASCs in response to core MFO NPs and core–shell MFO@BCZT MENPs under SMF and CMF conditions, we quantified the expression of key signaling molecules involved in mechanotransduction pathways. As established in the literature, cells perceive external mechanical stimuli through integrins, ion channels, cell-surface receptors, junction proteins, focal adhesion molecules, and cytoskeletal components, initiating intracellular signaling cascades that promote osteogenic differentiation.^115^ Figure S7 illustrates these mechanotransduction mechanisms and the intracellular pathways activated when magnetic NPs are attached to the cell membrane and exposed to an external magnetic field.^115–120^ Based on this framework, we assessed the expression of (1) extracellular signal-regulated kinase 1/2 (ERK 1/2), a downstream signaling molecule activated by integrins and PIEZO 1 channel-mediated signaling; (2) β-catenin, a key signaling molecule involved in pathways downstream of E-cadherins, Wnt-β-catetin pathway, and PIEZO channel activation; (3) phosphorylated focal adhesion kinase (FAK [pY397]), a marker of integrin receptor activation; and (4) bone morphogenetic protein 2 (BMP-2), an early osteogenic regulator whose expression is associated with PIEZO channel activation.^115–118^

As shown in Figure 9Ci, under SMF conditions, FAK [pY397] expression increased in both magnetized and nonmagnetized spheroids, with a significant increase in the magnetized groups. This suggests that the presence of NPs enhances mechanotransduction via integrin signaling. This amplified activation may explain the increased expression of osteopontin in magnetized spheroids cultured in SMF compared to nonmagnetized ones (Figure 9Ai). Under CMF, FAK [pY397] expression was significantly higher in spheroids treated with MFO@BCZT MENPs, supporting the hypothesis that the advanced osteogenesis observed under this condition is at least partially driven by integrin-mediated mechanotransduction.

For ERK 1/2, the analysis revealed increased expression in spheroids treated with MFO NPs under SMF, while levels remained relatively stable in spheroids treated with MFO@BCZT MENPs and in nonmagnetized controls (Figure 9Di and ii). However, no significant differences were found between magnetized and nonmagnetized groups under SMF. Under CMF conditions, however, a significant increase in ERK 1/2 expression was observed in magnetized spheroids treated with MFO NPs compared to nonmagnetic ones, further supporting the idea that the presence of NPs enhances the mechanical signal required to activate downstream signaling pathways. Moreover, differences were also found between the two magnetized spheroid groups, suggesting that spheroids treated with core MFO NPs have led to an earlier and higher expression of ERK 1/2 compared with spheroids treated with core–shell MFO@BCZT MENPs. Nevertheless, since ERK 1/2 activation can result from either integrin-mediated or PIEZO1 channel activation pathways, further analysis of PIEZO1 activation is necessary to infer its role in the osteogenic differentiation process.

PIEZO1 channels, which are stably expressed in mesenchymal stem cells, are mechanosensitive ion channels that play a key role in cellular signaling by converting mechanical stimuli into biochemical responses. They function primarily through calcium ion (Ca^2+^) signaling pathways, mediating Ca^2+^ influx and initiating downstream cascades involved in osteogenesis, including the upregulation of β-catenin and BMP-2, among others (Figure S7).^116^ Based on this mechanism, we assessed β-catenin expression as a potential downstream indicator. However, β-catenin was not detected under any of the conditions tested, implying that osteogenic differentiation in these spheroids likely proceeded through alternative pathways, independent of E-cadherin, Wnt/β-catenin, or PIEZO1-mediated signaling involving β-catenin. Conversely, BMP-2 expression was observed, and since its upregulation is specifically associated with PIEZO1 activation, these findings suggest that PIEZO1 signaling may still play a contributory role in directing hASC differentiation toward the osteogenic lineage.

As shown in Figure 9Ei and ii, BMP-2 was highly expressed in spheroids treated with MFO@BCZT MENPs under both SMF and CMF conditions, with a more pronounced expression under CMF. While BMP-2 expressions increased over time under SMF conditions in MFO@BCZT-treated spheroids, its expression decreased from day 7 to day 21 under CMF conditions.

Considering that BMP-2 expression is one of the first signals of mesenchymal stem cells’ commitment to the osteogenic lineage, its upregulation on day 7 under CMF conditions supported our conclusion that the combination of CMF exposure and the presence of core–shell MENPs accelerates osteogenic differentiation.^102,121^ As cells transition to mature osteoblasts, characterized by increases in osteopontin and osteocalcin and decreases in VEGF, COL I, and metabolic activity, a subsequent decline in BMP-2 expression is well documented in the literature, consistent with our observations.^102^ In contrast, the gradual increase in BMP-2 expression under SMF conditions (alongside lower levels of osteopontin and osteocalcin, increased metabolic activity, higher VEGF expression, and the presence of COL I at later time points) further supported a delayed osteogenic differentiation compared to CMF. Although intracellular Ca^2+^ levels were not directly measured over time, the upregulation of BMP-2 provides indirect evidence of calcium-dependent pathway activation. Future studies incorporating calcium imaging or quantitative ELISA-based calcium assays will be essential to confirm the enhanced intracellular content of calcium along the time, given its central role in initiating the osteogenic process. Additionally, the use of PIEZO1 conditional knockout should be considered to further elucidate the contribution of ion channels in osteogenic cell differentiation.^122^ Overall, the results presented herein suggest that osteogenic differentiation in both magnetized and nonmagnetized tissues under SMF and CMF conditions was likely mediated by the activation of integrin receptors and PIEZO1 channels.

### Biomineralization Analysis

To confirm the formation of bone-like tissue, mineralization was analyzed in spheroids after 7, 14, and 21 days of culture using SEM, EDS, and the OsteoImage mineralization assay. The results demonstrate that bone-like mineral formation occurred in both magnetized spheroid groups cultured under SMF and CMF conditions (Figure 10A and B). As expected, CMF stimulation led to more advanced mineralization, as evidenced by the presence of larger size minerals and more defined structures in SEM micrographs. The highest calcium-to-phosphorus (Ca/P) ratio was observed for spheroids treated with MFO@BCZT MENPs and cultured under CMF, reaching a Ca/P ratio of 1.04 on day 7, 1.39 on day 14, and 1.67 on day 21 (Figure 10Ci). In contrast, under SMF, they achieved a Ca/P ratio of 0.58 on day 7, 0.82 on day 14, and 1.59 on day 21, supporting our hypothesis of a slight delay in mineralization under SMF conditions. Given that the native Ca/P ratio of hydroxyapatite in bone is approximately 1.67, these results confirm that the combination of core–shell MFO@BCZT MENPs with CMF stimulation effectively promotes bone-like mineral formation, positioning CMF as a more potent driver of osteogenesis than SMF. As expected, mineralization in spheroids treated with core MFO NPs under CMF was less advanced than those treated with MFO@BCZT MENPs, reaching a Ca/P ratio of only 1.27 after 21 days in culture (Figure 10Cii). This finding further supports the superiority of MENPs, which enabled faster and more robust mineralization resembling native bone. Under SMF, spheroids treated with MFO NPs reached a Ca/P ratio of only 0.84 after 21 days.

**Figure 10:**
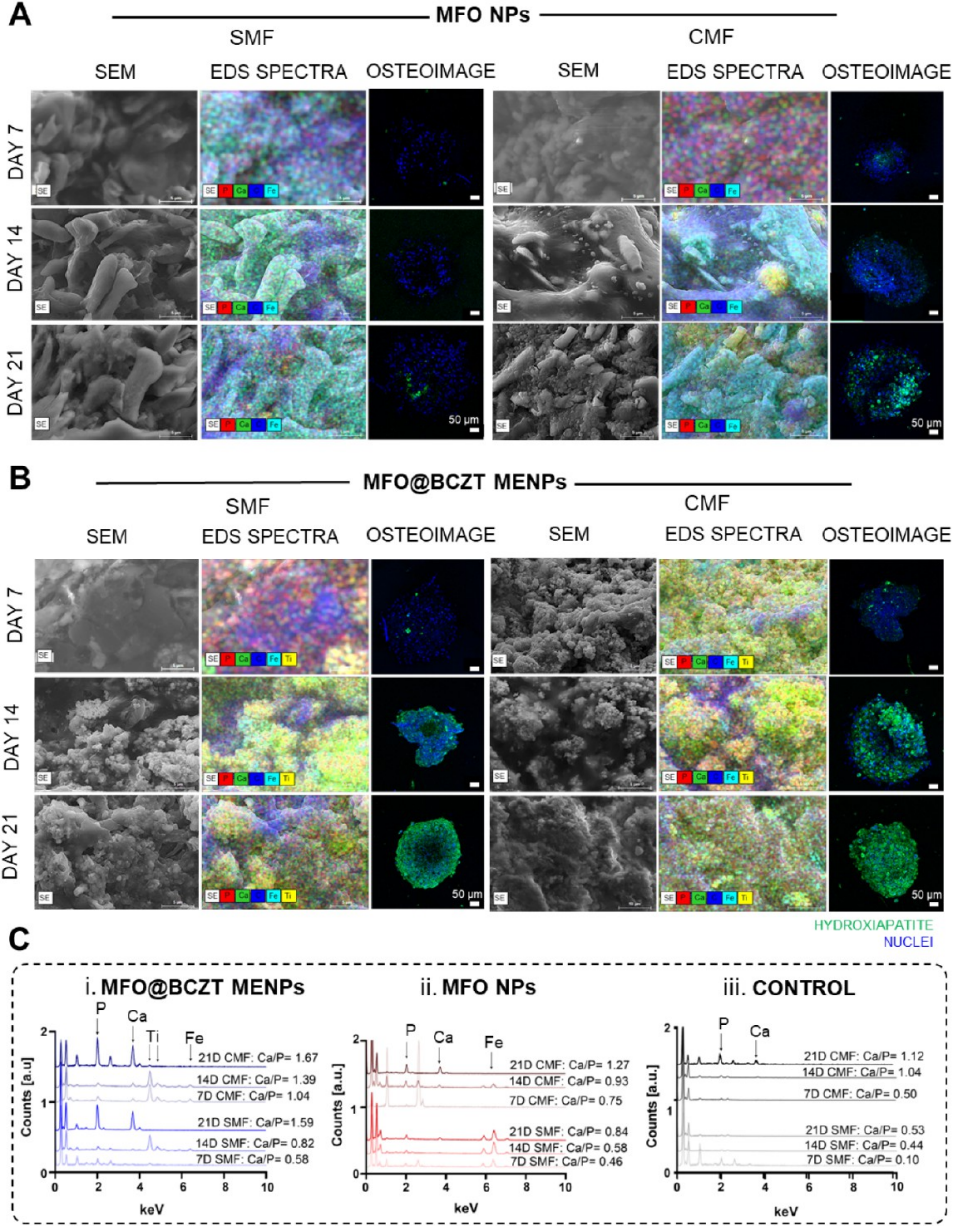
Mineralization ability of spheroids treated with MFO NPs and MFO@BCZT MENPs in response to SMF and CMF stimulation. (A and B) The analysis of bone-like mineral deposition for cells seeded with MFO NPs and MFO@BCZT MENPs using SEM, EDS spectra, and the OsteoImage staining assay. Scale bars: 5 μm for SEM micrographs and EDS micrographs; 50 μm for OsteoImage images. (C) The EDS spectra obtained for MFO@BCZT MENPs (i), MFO NPs (ii), and the control condition (without magnetic-based materials) (iii). Characteristic peaks indicating the presence of Ti and Fe for MFO@BCZT MENPs and Fe for MFO NPs are labeled. The absence of ferrous materials identified the control. Calculated Ca/P ratios are provided for each condition.

Consistent with previous findings based on osteogenic markers and cytokine secretion, nonmagnetized spheroids also underwent osteogenic differentiation when exposed to magnetic fields. However, the Ca/P ratios remained lower compared with magnetized groups, reinforcing the beneficial effect of combining magnetic NPs with magnetic field stimulation (Figures 10Ciii and S8). Specifically, nonmagnetized spheroids reached a Ca/P ratio of 0.53 under SMF and 1.12 under CMF after 21 days in culture. Although CMF stimulation alone promoted some hydroxyapatite deposition, the levels were still far from those achieved through the synergistic effect of CMF and MENPs.

### *In Vitro* Impact of ME Actuation for Remote Activation of Osteogenic Differentiation

To assess the *in vitro* impact of the remote ME effect on hASCs’ osteogenic differentiation, intracellular Ca^2+^ influx was measured after overnight exposure to SMF or CMF in magnetized and nonmagnetized cells. Intracellular Ca^2+^ levels are known to increase in response to electrical fields due to membrane depolarization, which opens voltage-gated Ca^2+^ channels (VGCCs) and stretch-activated Ca^2+^ channels (SACCs) and activates cell surface receptors.^15,123,124^ Regardless of the precise electrocoupled mechanism, these channels allow Ca^2+^ influx, triggering downstream pathways such as the Ca^2+^–calmodulin cascade that contribute to bone regeneration.^7,124^

As illustrated in Figure S9A, Ca^2+^ transients were observed in all conditions but were notably higher in cells internalizing MFO@BCZT MENPs compared with MFO NPs or unexposed cells. Furthermore, CMF enhanced the intracellular Ca^2+^ levels more efficiently than SMF. Quantification of cytosolic Ca^2+^ fluorescence (Figure S9B) confirmed that magnetic-field-induced strain was effectively transferred to the piezoelectric shell, leading to cell membrane depolarization. This experiment was designed as a proof of concept to confirm ME-induced Ca^2+^ influx after overnight stimulation rather than to provide a detailed quantitative comparison between SMF and CMF conditions. Nonetheless, the observed increase in Ca^2+^ signaling under CMF is consistent with earlier findings of enhanced osteogenic marker expression and mineralization at later time points. Future studies should incorporate ion channel blockers and real-time depolarization assays to further dissect the contribution and mechanism of intracellular ME stimulation.

## Conclusion

Although ME actuation holds great promise for biomedical applications, current ME nanomaterials face challenges related to biocompatibility and suboptimal piezoelectric performance, hindering their clinical application. In this work, we addressed this challenge by leveraging MENPs composed of a magnetostrictive manganese ferrite (MFO) core and a lead-free piezoelectric BCZT shell. We hypothesized that these MFO@BCZT MENPs, when internalized into hASCs and assembled into 3D spheroids, could enhance osteogenic differentiation under SMF or CMF stimulation. Unlike conventional systems, which require invasive electrodes or external power sources, magnetized spheroids enable remote-controlled stimulation with high spatiotemporal precision. Our results collectively demonstrate that the combination of CMF and MFO@BCZT MENPs significantly accelerated osteogenic differentiation, achieving a Ca/P ratio of 1.67 that closely resembled that of native bone apatite. In contrast, spheroids treated with magnetic core MFO NPs lacking the piezoelectric component exhibited delayed differentiation. Although SMF stimulation with MFO@BCZT MENPs also enhanced mineralization compared to that with MFO-treated controls, CMF proved to be the most effective modality. Overall, this study positions the integration of CMF stimulation with biocompatible, lead-free MFO@BCZT MENPs as a promising biomimetic strategy for bone regeneration. Compared to traditional electrical stimulation therapies, this approach eliminates the need for external power sources and wired electrodes, avoiding concerns related to electrical shocks, thermal injuries, and battery-related environmental concerns. Furthermore, magnetic field activation offers a safer alternative to ultrasound-based methods, which carry risks of tissue heating. Beyond bone repair, a self-powered and remotely controllable platform offers broad potential for other regenerative applications, including muscle and neural tissue engineering. By harnessing multistimuli responsiveness and mimicking native mechanotransduction pathways, this technology opens new avenues for the development of next-generation bioelectronic therapies.

## Methods

### Synthesis of Core MFO NPs and Core–Shell MFO@BCZT MENPs

MFO NPs and MFO@BCZT MENPs were synthesized by our partners from Tomsk Polytechnic University, following the protocols described elsewhere.^31^ Briefly, MFO NPs were produced by mixing 17.5 mM MnCl_2_·4H_2_O and 35 mM FeCl_3_·6H_2_O with 60 mL of deionized water under constant stirring. Afterward, 0.75 M of NaOH, previously dissolved in water, was added dropwise to the solution. The resulting solution was then sealed in an autoclave with a filling factor of 70% for 3 h at 200 °C. Subsequently, the precipitate was collected using an external magnetic field, washed extensively with deionized water until reaching a pH of 7, and dried under vacuum for 24 h. For the synthesis of MFO@BCZT MENPs, the ratio weights of BaCl_2_·2H_2_O, CaCl_2_, and ZrOCl_2_·8H_2_O were dissolved in distilled water and mixed under vigorous stirring. Next, pristine MFO NPs were added to the solution and sonicated in an ultrasonic bath for 5 min. Following this step, a TiCl_4_ solution was added dropwise into the solution, and the alkali concentration was adjusted to 16 mol L^–1^ by adding NaOH. The resulting solution was sealed in an autoclave with a filling factor of 60% for 24 h at 200 °C and washed with deionized water. Again, the NPs were recovered by magnetic separation and dried under a vacuum for 24 h.

### Morphological and Physical–Chemical Characterization of the Produced NPs

To perceive the internal structure of MFO NPs and MFO@BCZT MENPs, both NPs were imaged via HRTEM in a ThemisZ (Thermo Fisher Scientific, USA) electron microscope equipped with a Ceta 16 (Thermo Fisher Scientific, USA) CCD matrix. All of the samples were prepared in ethanol and dispersed in an ultrasonic bath before being drop-cast on holey carbon films mounted on copper grids. Images were acquired in the bright-field (BF)-HRTEM mode with an accelerated voltage set to 200 kV and analyzed using the VESTA-3 program. From these images, size measurements were performed, and the results were presented as mean ± SD. Afterward, to identify the materials elements at atomic resolution, high-angle annular dark-field scanning transmission electron microscopy (HAADF) was performed in the same equipment using a standard ThemisZ detector. The images were acquired in the annular dark field (ADF)-STEM mode. To complement this analysis, EDX mapping analysis was conducted by using a SuperX detector (Thermo Fisher Scientific, USA) on the preceding HAADF STEM images. The Fe/Mn ratio was calculated from two independent regions. Subsequently, to evaluate the crystalline structure of both NPs and to infer about the formation of the spinel MFO and the perovskite BCZT shell, XRD analysis was performed in a Shimadzu XRD-7000 diffractometer (Japan). In brief, samples were positioned on a holder and analyzed using Cu Kα radiation (λ = 0.154 nm) at 40 kV and 30 mA, in a 2θ ranging from 15° to 65° and a step size of 0.0143°. The phase composition from XRD patterns was calculated using the standard Rietveld refinement in Match software. XRD spectrograms were compared with PDF4+ files from the Powder diffraction file database. The phase composition was also calculated based on the HRTEM image (Figure 1), but thus obtained phase ratio for the single nanoparticle is apparently different from the average value provided by the Rietveld procedure. However, both values were close to each other. Besides this analysis, the crystalline structure was also evaluated by Raman spectroscopy using an InVia confocal dispersive Raman spectrometer (Renishaw, UK). The experiment was carried out using a laser beam with an excitation wavelength of 528 nm, which was focused on the sample with a 50× objective. The laser beam was limited to a maximum power of 95 mW to avoid sample overheating. To analyze the elemental composition as well as the chemical state of the atoms within the NPs, XPS and high resolution XPS were performed in a Thermo Fisher Scientific XPS NEXSA spectrometer mounted with a monochromated Al Kα Alpha X-ray source operating at 1486.6 Ev. The XPS spectra was acquired with a pass energy of 200 (eV) and energy resolution pf 1 eV from the surface area of 400 μm^2^, while the high-resolution spectra were acquired at the pass energy of 50 (eV) and energy resolution of 0.1 (eV). Magnetic measurements were then performed by using a pulsed magnetometer. Hysteresis loops were recorded at 300 K using a magnetic field strength ranging from 0 to 6.5 kOe, Additionally, ζ-potential measurements were conducted in a ZetaSizer Nano ZS instrument equipped with ZetaSizer software. Samples for zeta quantification were prepared by transferring 1 mL of a well-dispersed solution containing 1 mg mL^–1^ of each type of NPs in PBS into a zeta dip cell (DTS1070 folded capillary cell, Malvern). Data were analyzed by averaging three independent measures per formulation. In addition to ζ-potential analysis, the topography, surface potential (KPFM), magnetic structure, ferroelectric, and ME behaviors of the synthesized NPs were evaluated by scanning probe microscopy using NX-Hivac (Park Systems, South Korea) and Ntegra Aura (NT-MDT, Netherlands) instruments. The KPFM was studied with a two-pass technique under a noncontact regime at the fundamental resonance of the Budget sensors Multi75E-G cantilevers. The magnetic structure of NPs was investigated by means of MFM. The MFM measurements were performed using magnetic cantilevers Multi 75 M-G (Budget Sensors, Bulgaria) with a spring constant of 3 N/m and a resonance frequency of 75 kHz. To study the ferroelectric and ME performance of NPs via PFM, conductive Cr/Pt-coated cantilevers were used with a spring constant of 48 N/m and a resonance frequency of 190 kHz was used. To avoid the influence of the tip wear on the quality of the results, a new cantilever was used for each single MFM, KPFM, and PFM scan. Finally, to confirm the piezoelectric properties of the MENPs under varying external magnetic fields, PFM was employed with a DC bias voltage and an external in-plane SMF (500 Oe) applied to switch the direction of the polarization and to characterize the ME coupling, respectively. The direct magnetoelectric coefficient (α_ME_), which reflects apparent coupling between piezoelectric and magnetostrictive phases of NPs, was estimated as follows:^71^

1 $$\alpha_{\mathrm{ME}}=\frac{\Delta E}{\Delta H}=\frac{\Delta V/t}{\Delta H},\left[V{\mathrm{cm}}^{-1}{\mathrm{Oe}}^{-1}\right]$$

where Δ*H* is the applied magnetic field and Δ*E* is the electric field change, which is defined as the coercive-voltage change (Δ*V*) divided by the piezoelectric shell thickness (*t*).

### Stability of MFO NPs and MFO@BCZT MENPs in Culture Medium

The *in vitro* stability of the NPs in the culture medium was evaluated by monitoring changes in the absorbance of resuspended and nonresuspended NPs, as well as changes in ζ-potential over time. These procedures followed adapted protocols described in the literature.^79,125^ To quantify the absorbance, lyophilized MFO NPs and MFO@BCZT MENPs were weighed and mixed with 70% (v/v) ethanol for overnight sterilization on a rotator disk at 30 rpm. On the next day, the NPs were centrifuged at 300 g for 5 min, washed three times with Dulbecco’s phosphate-buffered saline (dPBS) to remove the ethanol residues, and resuspended in the culture medium consisting of Minimum Essential Alpha Medium (α-MEM) supplemented with 1% antibiotic–antimycotic and 10% (v/v) FBS. Then, 500 μL of these solutions were transferred to Eppendorf’s, in six replicates, and incubated for 24 h at 37 °C in a humidified incubator with 5% CO_2_. Subsequently, 100 μL of supernatant from three replicates were transferred into a 96 Helma Quartz Microplate without homogenization, while the other triplicates were resuspended before being placed in the microplate. The absorbance was recorded in a Synergy HTX Biotek microplate reader at 480 nm. This wavelength was determined before analysis by recording the absorbance spectra of both MFO NPs and MFO@BCZT MENPs in a Helma Quartz Microplate between 250 and 700 nm (Figure S10). To measure the ζ-potential, 1 mL of NP solution in the culture medium was transferred to Eppendorf’s tubes and maintained at 37 °C in a humidified incubator with 5% CO_2_ for 21 days. On days 0, 1, 3, 7, 14, and 21, the ζ-potential was measured using a DLS system integrated with a ZetaSizer Nano ZS instrument. Measurements were performed using DTS1070 zeta cells (Malvern), and the temperature was set to 37 °C to replicate the *in vitro* conditions. Medium changes in the Eppendorf’s tubes were carried out every 3 days by placing a magnet beneath each tube to prevent pH alterations in the culture. For both experiments, a plain culture medium was used for control. Three independent replicates were made per formulation, and results were presented as mean ± SD.

### hASC Isolation, Culture, and Characterization

Cell culture experiments were conducted using hASCs isolated from subcutaneous adipose tissues obtained during a liposuction procedure. The adipose tissues were obtained under a cooperation agreement between the COMPASS Research Group situated in CICECO, Aveiro Institute of Materials, University of Aveiro, the Centro Hospitalar do Baixo Vouga (Aveiro, Portugal), and Hospital da Luz (Aveiro, Portugal), after approval by the Competent Ethics Committee (CEC, agreement number 10052020), with informed consent from all parties and from all participants involved. The isolation of hASCs followed a previously reported protocol.^113^ Briefly, the collected adipose tissue was transported to the university facilities in dPBS supplemented with 10% (v/v) penicillin–streptomycin and washed several times until the aqueous solution became clear. Then, the lipoaspirates were digested with 0.1% (w/v) collagenase type I during 45 min at 37 °C, centrifuged at 1200 rpm for 10 min, washed with dPBS, and centrifuged again. The resultant pellet was resuspended in α-MEM supplemented with 1% antibiotic-antimycotic and 10% (v/v) FBS and transferred to appropriate t-flasks. The cells were maintained at 37 °C in a humidified incubator with 5% CO_2_, with the medium being replaced every 3 days. To validate the stemness phenotype of the isolated hASCs, cells were incubated with fluorescently labeled monoclonal antibodies against CD31, CD34, CD73, CD90, and CD105 markers and analyzed with flow cytometry (BD Accuri C6 Plus flow cytometer, BD Biosciences). Cells at a maximum passage of 4 were used for cell differentiation studies.

### Magnetic Cell Internalization

Magnetic cell internalization was carried out by seeding 1 × 10^4^ cells per well into 48-well plates with 200 μL of α-MEM for 24 h. MFO NPs and MFO@BCZT MENPs were sterilized as previously described and used to prepare solutions in the culture medium with concentrations spanning 0, 1, 3, and 6 mg of NPs per 10^6^ cells. To ensure proper dispersion in the culture medium, all solutions were sonicated in an ultrasonic bath (P 120 H, 12.75 L, Elmasonic) for 5 min at 37 °C before being added to the cells. Following sonication, the medium cultured with cells was replaced by 200 μL of the freshly prepared NP solutions. Internalization studies were conducted for 4, 6, 8, 12, and 24 h and monitored using optical contrast light microscopy (Primostar, Carl Zeiss, Germany). hASCs that did not undergo NP internalization were used as controls. Their preparation followed the same procedure, with the exception that the incubation step with the NPs was replaced by the addition of 200 μL of fresh medium.

### Assessment of Cellular Uptake of the NPs

The cellular uptake of MFO NPs and MFO@BCZT MENPs was assessed by comparing the absorbance of the NPs within the cells to those remaining in the culture medium. At 4, 6, 8, 12, and 24 h, the medium containing the noninternalized NPs was collected into Eppendorf’s tubes, centrifuged at 300g for 5 min, and washed with dPBS. Simultaneously, magnetized cells were washed thrice with dPBS and incubated with 200 μL of 0.1% (v/v) Triton X-100 solution for 1 h at 37 °C to permeabilize the cells. Following this incubation, the wells were mechanically scraped to detach the magnetized cells, and the absorbance was measured at 480 nm using a microplate reader (Synergy HTX, Biotek Instruments, USA), as determined above. Wells containing Triton X-100 and nonmagnetized cells served as controls. The experiment was conducted in triplicate, and results were processed by calculating the ratio of the absorbance registered for the cells divided by the total absorbance of the medium to the cells. Results were converted to percentages and presented as the mean ± SD.

### Assessment of Cells’ Lysosomal Expression

To assess whether NP uptake occurred via endocytosis, magnetized and nonmagnetized hASCs were stained with LysoTracker to label lysosomes. Cells were seeded and incubated with both types of NPs as described previously and allowed to internalize for 24 h, as these conditions were found to be optimal for uptake. Subsequently, cells were incubated with 200 μL of prewarmed culture medium containing 60 nM LysoTracker for 2 h at 37 °C, following the manufacturer’s recommendations. After incubation, cells were washed with dPBS, fixed in 4% (w/v) formaldehyde in PBS for 2 h at RT, and counterstained with DAPI (1:500 in PBS) for 5 min at RT. Samples were imaged in a confocal laser microscope (LSM 900, Carl Zeiss) equipped with a 63× oil objective. Lysosomal fluorescence was quantified using ImageJ software via the IntDensity function, and results were normalized against the control group and expressed as a percentage.

### Cytotoxicity, Morphology, and Cellular Proliferation Analysis of Magnetized Cells

The cytotoxic profile of MFO and MFO@BCZT NPs on cells was initially visualized by a live/dead fluorescence assay following the manufacturer’s recommendations. Briefly, hASCs were seeded at a density of 1 × 10^4^ cells/per well in ibidi μ-Slide 8-well plates, incubated with 0, 1, 3, and 6 mg of NPs per 10^6^ cells on the following day, and treated with a fresh culture medium 24 h later. On days 1, 3, and 7, the culture medium was removed, and cells were incubated with the live/dead reagents (Calcein AM and PI at a ratio of 2:1000 and 1:1000, respectively, in dPBS) at 37 °C for 30 min, protected from light. The live and dead cells were imaged in a confocal laser microscopy coupled with a 10× objective, with an excitation wavelength of 488 nm and an emission wavelength of 570 nm. After live/dead analysis, all the samples were rinsed extensively with dPBS and fixed with 4% (w/v) formaldehyde solution in PBS for 2 h at RT. After fixation, cells were incubated with 200 μL of 0.1% (v/v) Triton-X100 in ultrapure water for 10 min, washed once with PBS, and then blocked with 5% FBS (v/v) in PBS for 1 h at RT. After some washing steps, samples were incubated with red phalloidin at a 1:40 ratio in dPBS for 20 min at RT to stain the actin filaments and with DAPI at a 1:1000 ratio in dPBS for 5 min at RT to stain the nuclei, before being visualized under confocal microscopy.

Additionally, cells’ viability was quantified using the MTS colorimetric assay, also following the manufacturer’s recommendations. In brief, cells were treated as described above, with the exception that they were cultured in 48-well plates. At the same time points, the culture medium was replaced by a mixture of 200 μL of dPBS and 40 μL of the reagent solution, in agreement with the manufacture’s recommendations. After 4 h of incubation at 37 °C under dark conditions, the absorbance was read at 490 nm using a microplate reader. Wells containing solely an MTS mixture were used as controls. All conditions were normalized to the control group (nonmagnetized cells), which was set at 100% viability. A total of four replicates were considered for each formulation. Following MTS quantification, all the samples were washed with PBS, incubated with 200 μL of 0.1% (v/v) Triton X-100 in ultrapure water for 1 h at 37 °C, and frozen at −80 °C until analysis. Upon collecting all the time points, samples were thawed at RT, sonicated in an ultrasonic bath for 5 min (37 Hz, sweep field, 60% potency), and frozen at −20 °C to induce cellular disruption. The process was repeated twice before determining the DNA content using the Quant-iT PicoGreen dsDNA Assay Kit in accordance with the manufacturer’s instructions. The amount of DNA present in the samples was then inferred against the calibration curve prepared according to the kit guidelines.

### Preparation of Magnetized and Nonmagnetized Spheroids

Upon the internalization of MFO NPs and MFO@BCZT MENPs within cells, magnetized cells were detached using 200 μL of TrypLE Express for 5 min at 37 °C. Following the inactivation of TrypLE Express with the double quantity of the culture medium, cells were centrifuged at 300 g for 5 min and seeded on an ultralow adhesion 96-well plate (Corning) at a cell density of 1 × 10^4^ cells/well/150 μL of culture medium. To encourage the self-aggregation of cells into 3D spheroids, seeded cells were first centrifuged at 500 g for 10 min and then magnetically attracted by placing a neodymium disc magnet (Ø 20 mm, height 2 mm, 22.6 N strength, and standard N35 magnetization) at the bottom of each well for 3 days, as illustrated in Figure 7Ai. For the nonmagnetic spheroids, pristine hASCs were seeded on ultralow adhesion 96-well plates, centrifuged, and incubated for 3 days without magnetic stimulation.

### Spheroid Encapsulation within GelMA Hydrogel

After 3 days of culture, magnetized and nonmagnetized spheroids were encapsulated within GelMA hydrogels and fixed to glass coverslips. Before the experiment, GelMA was synthesized by reproducing the described protocol, and glass coverslips were functionalized with methacrylated groups following an adapted protocol.^126,127^ Briefly, the coverslips were treated three times with plasma (Plasma System ATTO, Electronic Diener) for 5 min at 0.2–0.4 mbar and 30 V and then immersed in a solution prepared with 2% (v/v) of 3-(trimethoxysilyl)­propyl methacrylate (TMPSM) in toluene for 24 h. After this time, the coverslips were extensively washed with 70% ethanol and dried inside a flow chamber for sterilization. On the encapsulation day, lyophilized GelMA was dissolved in a solution of 0.5% (w/v) 2-hydroxy-4′-(2-hydroxyethoxy)-2-methylpropiophenone (Irgacure) in PBS to achieve a final concentration of 10% (w/v). Afterward, each spheroid was transferred from the 96-well plate to a 1 mL Eppendorf tube filled with 200 μL of GelMA precursor solution and then moved with 5 μL of GelMA to the functionalized coverslip. Four hydrogels, each of which containing one spheroid encapsulated within it, were placed separately on the same coverslip and photopolymerized using an Omnicure S20000 UV Lamp (Sarspec) positioned 4 cm from the lamp collimator, for 60 s at 2.5 w/cm^2^, as illustrated in Figure 7Aii.

### Magnetic Field Stimulation

To assess the effect of the magnetic field over the stem cell spheroids (magnetized and nonmagnetized), samples were exposed to either SMF or CMF. For the SMF, coverslips carrying the hydrogels with the spheroids were cultured in nontreated 24-well plates and stimulated continuously by placing a neodymium disc magnet (Ø 20 mm, height 10 mm, 108 N strength, and standard N42 magnetization) (Supermagnete) below each sample well. For the experiments requiring a CMF, a cyclic lab-made device was fabricated (Figure S11). The apparatus consisted of a 28 cm poly­(vinyl alcohol) (PVA) bar with one neodymium disc magnet, previously described, affixed to each end. The bar was connected to a rotary motor, enabling the continuous circular movement of the magnets. To position the samples at the appropriate height relative to the rotating bar, 3D-printed supports were designed by using the Sharp3D software and printed on a Flashforge Creator 3 V2 3D printer (Beeverycreative). Using the same equipment, 3D-printed molds were designed to hold four coverslips, ensuring that all samples align in the circular trajectory made by the rotating magnets. To fit within standard Petri dishes, the molds were designed with an outer diameter of 6 cm and an inner diameter of 5 cm. For coverslip placement, each mold included four cylindrical holders (1.6 cm diameter × 0.5 cm height) in their inner area. To produce negative replicas, the printed molds were then cast in polydimethylsiloxane (PDMS) at a 10:1 (w/w) molar ratio of prepolymer to curing agent and cured overnight in an oven at 37 °C. The resulting PDMS countermolds were removed, and before the assay, they were sterilized in ethanol overnight and dried inside a laminar flow chamber. Afterward, they were placed inside sterilized Petri dishes and positioned atop the printed supports. The frequency of the rotary motor was set to 71 mHz. In both SMF and CMF scenarios, samples were positioned 3.5 cm away from the magnets, detecting a magnetic field intensity of 13 mT, measured with a Gaussmeter (Tesla Meter). These parameters and magnets (108 N) were chosen based on our earlier study demonstrating enhanced bone formation *in vivo* following cyclic magnetic stimulation, with no adverse effects on implanted tissues or animal health.^128^ In addition, the selected motor frequency lies within the biologically relevant low-frequency range (<1 Hz) known to stimulate osteogenic differentiation without causing structural damage to soft tissues. Previous studies have shown that such low-frequency magnetic fields activate mechanotransduction pathways, promote osteoblast activity, and enhance mineral deposition, whereas lower field intensities and frequencies are more often associated with adipogenic responses.^129^ Together, these considerations provide a strong rationale for using these parameters as the basis for future *in vivo* studies evaluating the performance, safety, and fate of the NPs. All the conditions were cultured with 750 μL of culture medium and maintained in culture for 7, 14, and 21 days.

### *In vitro* Assessment of Osteogenic Differentiation Enhanced by Magnetic Stimulation

#### Assessment of Cell Viability, Metabolic Activity, and Proliferation

The viability of encapsulated spheroids exposed to magnetic fields was assessed after 7, 14, and 21 days using a live–dead fluorescence assay, following the manufacturer’s guidelines. At each time point, the hydrogels were detached from the coverslips and stained with a mixed solution of calcein AM and PI, following the procedure above-described. In addition, viability was quantified by using the MTS colorimetric assay. In brief, the four hydrogels per coverslip were detached and transferred to a single Eppendorf tube. The hydrogels in the tube were washed with dPBS and digested with 500 μL of 0.1% (w/v) collagenase type IV solution in dPBS for 2 h at 37 °C at 30 rpm in an orbital shaker to release the encapsulated spheroids. The released spheroids were then incubated with the mixture of MTS and analyzed, as described earlier. Following MTS measurements, the samples were also stored for DNA quantification and processed by following the procedure described earlier.

#### Osteopontin, Osteocalcin, and Collagen Immunofluorescence Detection

The secretion of osteopontin, osteocalcin, and COL I in response to SMF and CMF stimulation was analyzed through immunofluorescence staining. At each time point, samples (without being digested by collagenase solution) were fixed with 4% (w/v) formaldehyde solution for 2 h at RT, washed with dPBS, and stored at −4 °C until analysis. For staining, samples were permeabilized for 10 min in 200 μL of 0.1% (v/v) Triton-X100 solution in ultrapure water at RT and blocked for 1 h in a solution of 5% (v/v) FBS in dPBS. For osteopontin detection, samples were incubated overnight at 4 °C with the primary rabbit antihuman osteopontin antibody (Merck Millipore, 1:100 in 5% (v/v) FBS in dPBS). For osteocalcin and COL I detection, samples were incubated overnight at 4 °C with the primary rabbit antihuman osteocalcin antibody (at a 1:100 ratio in 5% (v/v) FBS in dPBS) and the Collagen I mouse monoclonal antibody (at a 1:200 ratio in 5% (v/v) FBS in dPBS). After sequential washing with dPBS, osteopontin-stained samples were incubated for 1 h at RT with the secondary antirabbit Alexa Fluor 488 antibody (1:400 in 5% (v/v) FBS in dPBS). Osteocalcin and COL I-stained samples were incubated with the secondary antirabbit Alexa Fluor 488 antibody (at 1:400 in 5% (v/v) FBS in dPBS) and the antimouse Alexa Fluor 647 antibody (1:400 in 5% (v/v) FBS in dPBS). All samples were counterstained with DAPI (1:1000 in dPBS) for 5 min at RT and observed under confocal microscopy using a 10× objective.

#### ELISA Immunoassay for Secretome Detection

During the 21 days of the assay, the secretome of encapsulated spheroids within GelMA hydrogels exposed to SMF and CMF was collected in Eppendorf’s tubes and stored at −80 °C until analysis. The expression of VEGF, osteopontin, ERK 1/2, beta-catenin, and FAK [pY397] was quantified by ELISA kits using the Human VEGF ELISA Kit (catalog no. ab222510, Abcam), Human Osteopontin ELISA Kit (catalog no. ab269374, Abcam), ERK1/2 ELISA Kit (catalog no. ab17664, Abcam), beta-catenin (Total) Human ELISA Kit (catalog no. KHO1211, Invitrogen), and FAK [pY397] ELISA Kit (catalog no. KHO0441, Invitrogen), respectively. All the kits were handled according to manufacturer’s instructions and read at the specified wavelength using a microplate reader. The Micro BCA Protein Assay Kit (catalog no. 23235, Thermo Fisher Scientific) was used to normalize the ERK 1/2 results, and the FAK (Total) Human ELISA Kit (catalog no. KHO043, Invitrogen) was used to normalize the expression of FAK [pY397]. As controls, wells containing the culture medium that were in contact with plain GelMA hydrogels were used.

### Analysis of Hydroxyapatite Deposition

The deposition of hydroxyapatite-like minerals by cells in response to SMF and CMF conditions was assessed after 7, 14, and 21 days of culture using the OsteoImage Mineralization Assay kit (Catalog # CABRPA-1503, VWR) and EDS. In brief, samples were washed with dPBS and fixed with 4% (v/v) formaldehyde solution for 2 h at RT. For the OsteoImage assay, samples were washed with 1× wash buffer provided in the kit and incubated with 200 μL of the staining reagent (1:100 in dilution buffer) for 30 min in the dark. After incubation, samples were rewashed with 1× wash buffer and counterstained with DAPI (1:500 ratio in PBS) for 5 min at RT. Imaging was performed on a confocal microscopy using a 10× objective. For EDS analysis, the spheroids were carefully removed from the hydrogels with the aid of a needle and washed with distilled water. Afterward, they were mounted on aluminum stubs covered with carbon conductive tape and sputtered with a thin film of carbon (K950X Turbo-Pumped Carbon Evaporator). Hydroxyapatite nodules were visualized using a Scanning Electron Microscopy (SEM, SU70, Hitachi) equipment coupled with an EDS detector (QUANTAX 400, Bruker). The equipment operated at an electron intensity of 15 kV and a working distance of 10 mm. The ratio of Ca and P peaks was determined from the EDS spectra using Esprit software.

### Intracellular Calcium Measurements

The hASCs were initially seeded and internalized with MFO and MFO@BCZT NPs, as previously described. After 24 hours of internalization, magnetized and nonmagnetized cells (control) were washed with dPBS and exposed to CMF and SMF overnight. To detect intracellular Ca^2+^ ions, a solution of 2 μM of Fluo-4 AM (MedChem Express) with 0.02% of Pluronic F-127 (Laborspirit) was prepared in α-MEM without FBS, following adapted protocols.^15,123^ To stain the nuclei of living cells, Hoechst 33342 (Alfagene) was added to the solution at a ratio of 1.6 μL: 400 μL of culture medium. Cells were then incubated with 200 μL of the solution containing Fluo-4 AM/Hoechst 33342 for 45 min in the dark at RT. After incubation, cells were washed with α-MEM without FBS and imaged under confocal microscopy using the 63× oil objective. The fluorescent signal of intracellular Ca^2+^ was quantified using ImageJ software using the IntDensity function.

### Statistical Analysis

All statistical analyses were analyzed using GraphPad Prism v10.1.2 software or the ImageJ software (Java 1.8.0_172). Results were expressed as mean ± SD, with significance set at *p* < 0.05 (****p < 0.0001; ***p < 0.001; **p < 0.01; *p < 0.05). The results marked with an asterisk (*) were considered statistically significant.

## Supplementary Material



## Notes

High-resolution XPS analysis identifying the surface chemical states of MFO and MFO@BCZT NPs; flow cytometry analysis of hASCs; confocal images and quantification of LysoTracker probe staining of lysosomes in cells treated with different concentrations of MFO and MFO@BCZT NPs, as well as in untreated control cells; confocal DAPI/Phalloidin staining images of nonmagnetized and magnetized cells exposed to various concentrations of both NP types; confocal images showing osteogenic marker expression (collagen I, osteocalcin, and osteopontin) in magnetized spheroids treated with core MFO NPs and core–shell MFO@BCZT MENPs, as well as in nonmagnetized spheroids, all evaluated under SMF and CMF conditions; schematic illustration of typical signaling pathways activated when magnetic NPs are attached to the cell surface and exposed to magnetic fields; SEM, EDS, and OsteoImage staining analyses of nonmagnetized spheroids under SMF and CMF conditions; confocal images and quantification of intracellular Ca^2+^ influx in magnetized and nonmagnetized hASCs after overnight exposure to SMF and CMF; UV–Vis absorbance spectra of MFO NPs and MFO@BCZT MENPs; and a photograph of the custom-built apparatus developed to generate CMF conditions (PDF)
