# Supplementary material for: Osteogenic Differentiation Triggered by Intracellular Magnetoelectric Stimulation of Core–Shell Nanotransducers under Remotely Applied Magnetic Fields
Source: ACS Nano. 2025 Dec 5;19(50):42022–45. doi: 10.1021/acsnano.5c10509 (PMC12818836; doi:10.1021/acsnano.5c10509)
Supplement: Supplementary file 1 [file nn5c10509_si_001.pdf]

Osteogenic differentiation triggered by intracellular magnetoelectric stimulation of core-shell nanotransducers under remotely applied magnetic fields

Maria C. Mendes[1], Elisa A.G. Martins[1], Roman V. Chernozem[3], Polina V. Chernozem[3], Catarina C. Custódio[1], Roman A. Surmenev[2,3], Andrei L. Kholkin[4], Ana S. Silva[1], João F. Mano[1*]*

[1]Departament of Chemistry, CICECO, University of Aveiro, Campus Universitário de Santiago, 3810-193 Aveiro, Portugal.

[2]Physical Materials Science and Composite Materials Centre, [3]International Research & Development Center "Piezo- and Magnetoelectric Materials", Research School of Chemistry & Applied Biomedical Sciences, National Research Tomsk Polytechnic University, Tomsk 634050, Russia

[4]Department of Physics & CICECO, University of Aveiro, Campus Universitário de Santiago, 3810-193 Aveiro, Portugal

Supporting Information

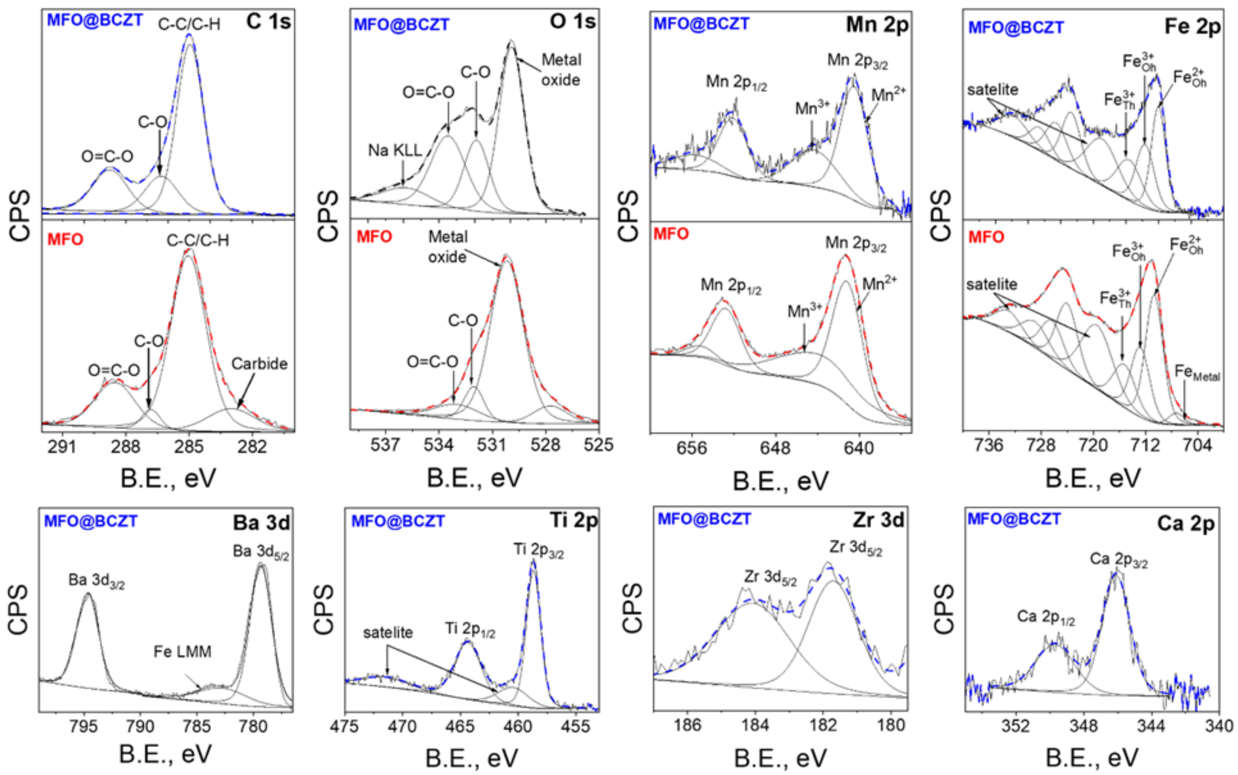

Figure S1. Identification of surface chemical states of MFO and MFO@BCZT NPs via high-resolution XPS analysis.

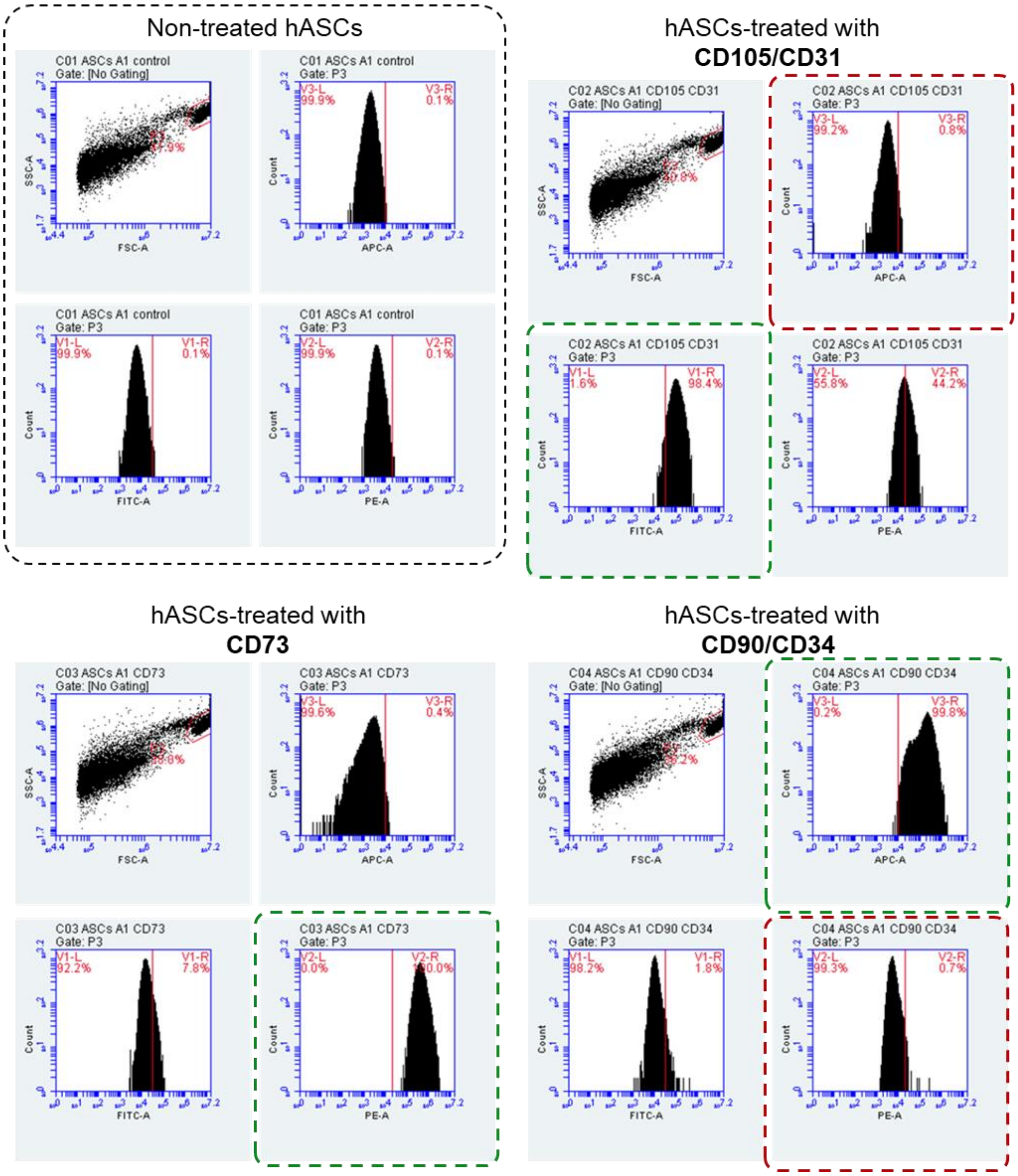

Figure S2. Flow cytometry analysis confirming the stemness phenotype of hASCs. The hASCs exhibited negative expression of the endothelial markers CD31-APC and CD34-APC (indicated in red), and positive expression of the mesenchymal stem cell markers CD105-FITC, CD73-PE, and CD90-APC (indicated in green).

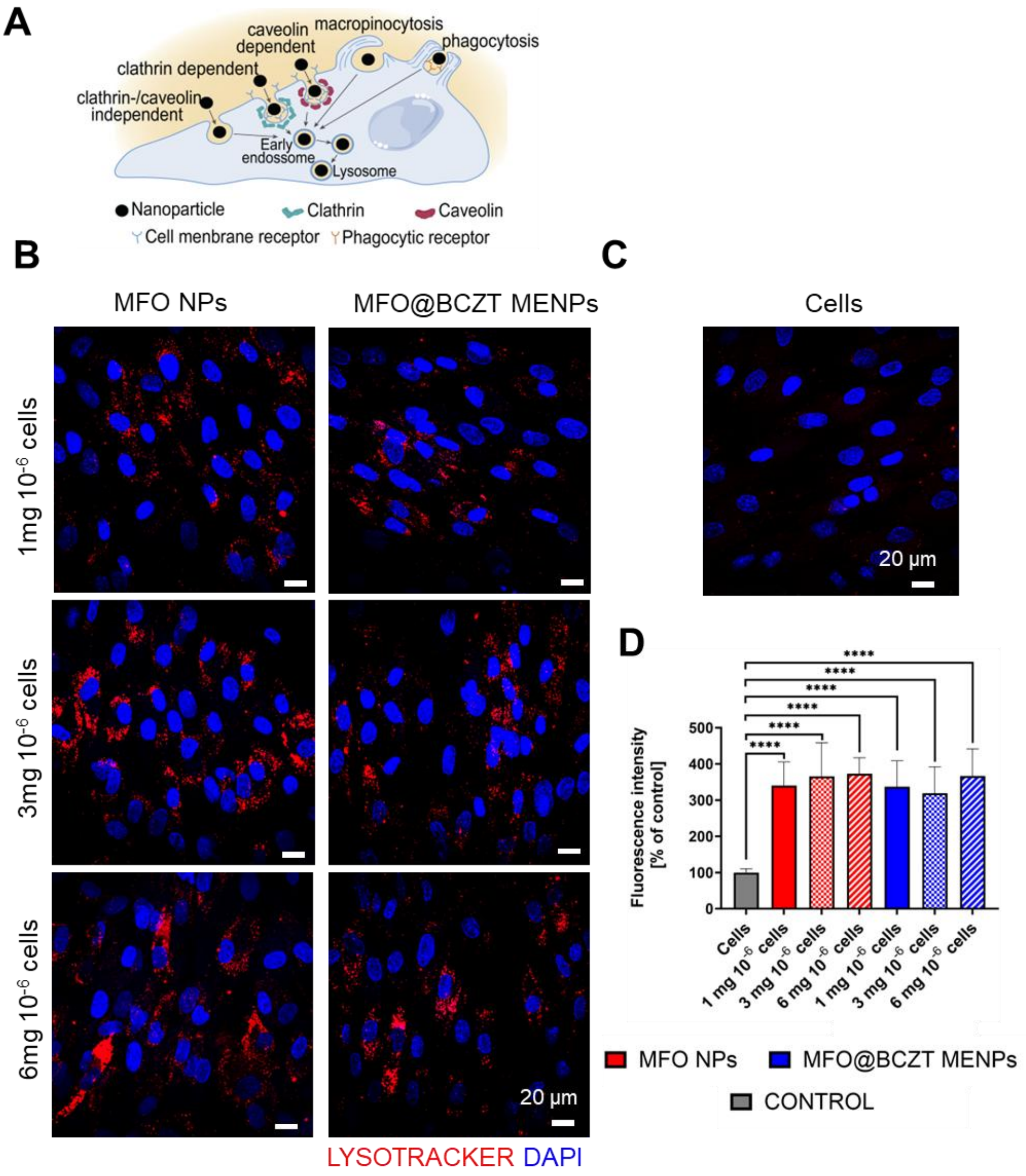

Figure S3. Lysotracker probe staining and quantification. A) Schematic overview of the different endocytic pathways involved in NPs internalization and their subsequent enclosure within lysosomes, as supported elsewhere.[1,2] Illustration created with Adobe Illustrator B) Representative confocal images of the lysotracker staining showing lysosomes (red) and nuclei (blue) at varying NP concentrations, and in C) cells without NPs upon 24 h of NPs internalization. Images were acquired under 63x oil objective. Scale bar: 20 µm. D) Quantification of fluorescence intensity using the IntDensity feature in ImageJ, with results normalized as a percentage of the control. Data are presented as mean ± SD (n =6).

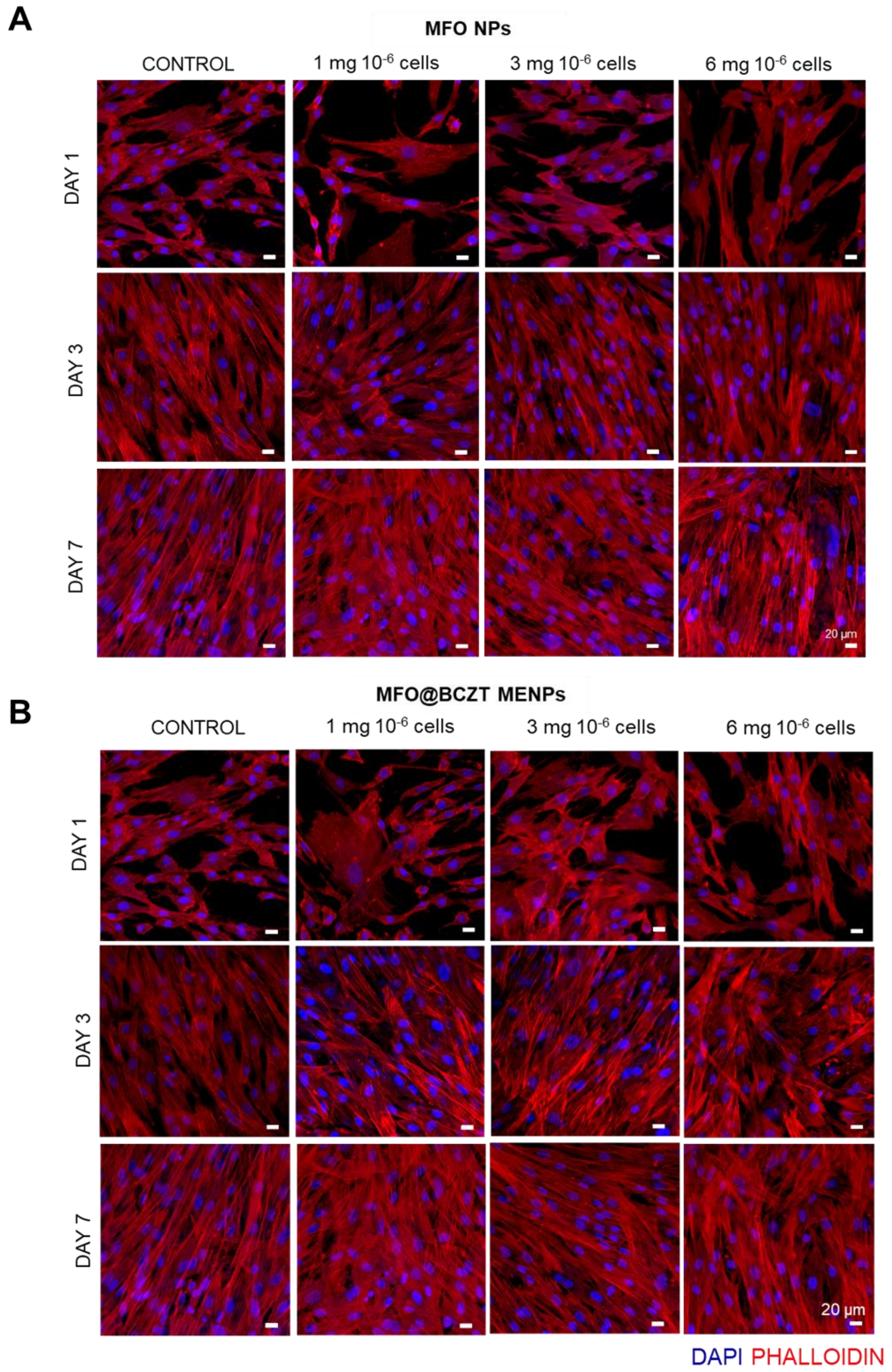

Figure S4. Morphological characterization of magnetized hASCs exposed to varying concentration of NPs. A) Representative confocal laser microscopy images depicting hASCs that have internalized MFO NPs, and B) those that have internalized MFO@BCZT MENPs. Non magnetized cells served as controls for both formulations. Cultures were maintained for up to 7 days. Images were acquired using a 10x objective Scale bar: 20 µm.

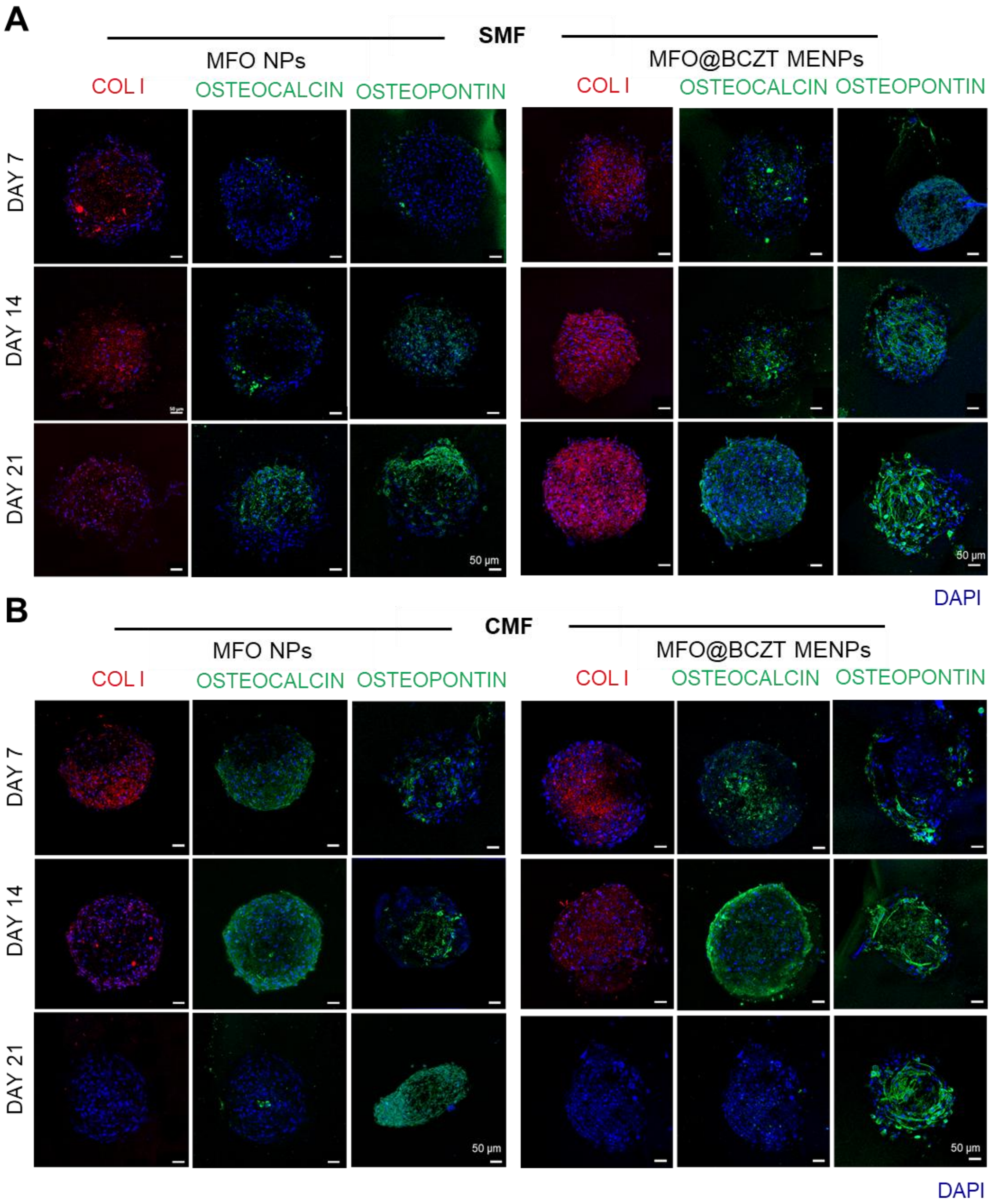

Figure S5. Osteogenic markers in magnetized spheroids treated with core MFO NPs and core-shell MFO@BCZT MENPs. Representative confocal images showing the expression of COL 1 (red), osteocalcin (green) and osteopontin (green), with cell nuclei counterstained with DAPI (blue). Magnetized tissues were evaluated up to 21 days post spheroid formation and compared against A) SMF or B) CMF culture conditions. Images were acquired using a 10x objective. Scale bar: 50 µm.

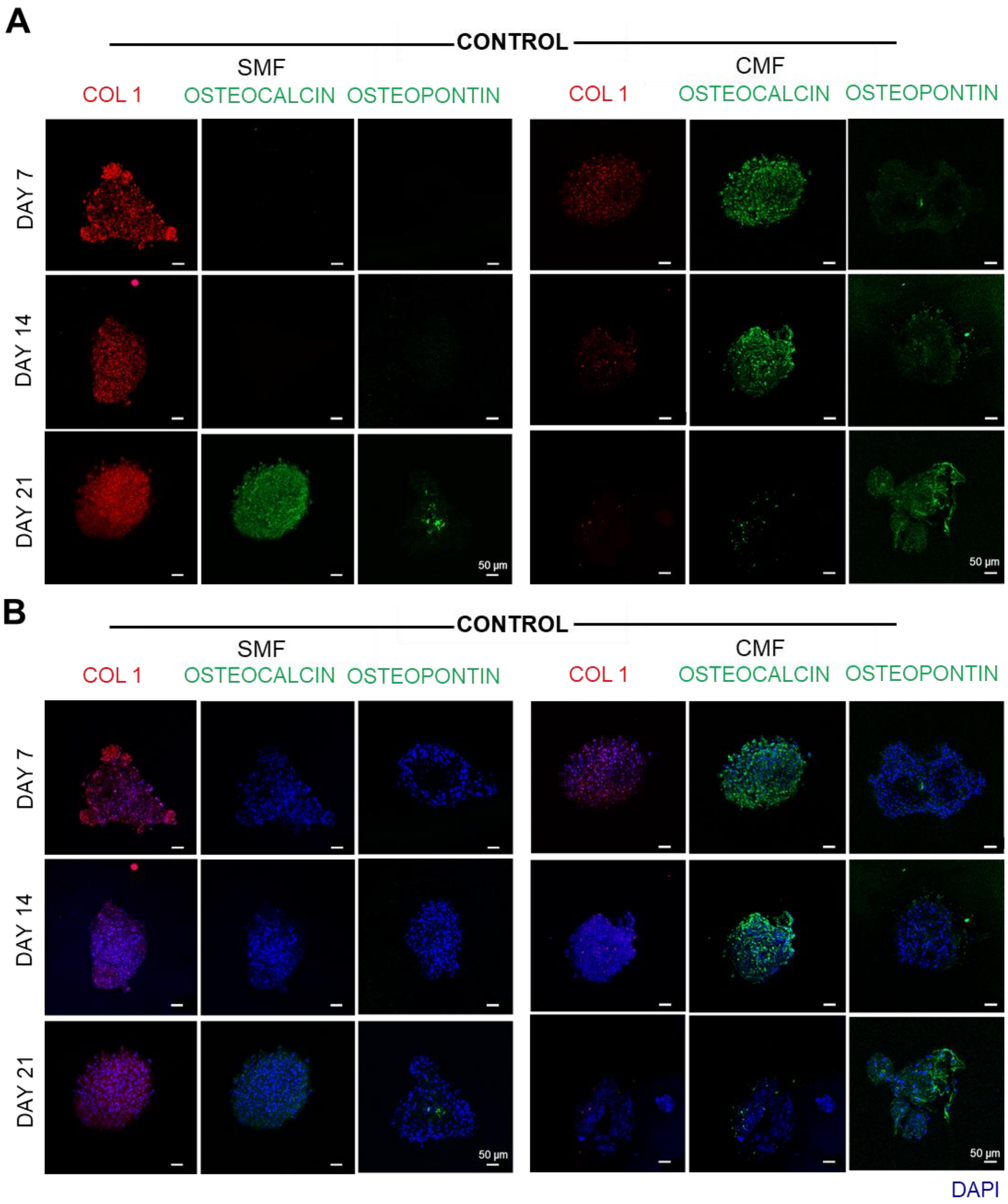

Figure S6. Detection of osteogenic markers in non-magnetized spheroids culture and SMF and CMF conditions. A) Representative confocal images showing individual expression of COL 1 (red), osteocalcin (green) and osteopontin (green). B) Merged images with DAPI (blue) to visualize cell nuclei. Non-magnetized tissues were evaluated up to 21 days post spheroid formation and compared against SMF or CMF conditions. Images were acquired using a 10x objective. Scale bar: 50 µm.

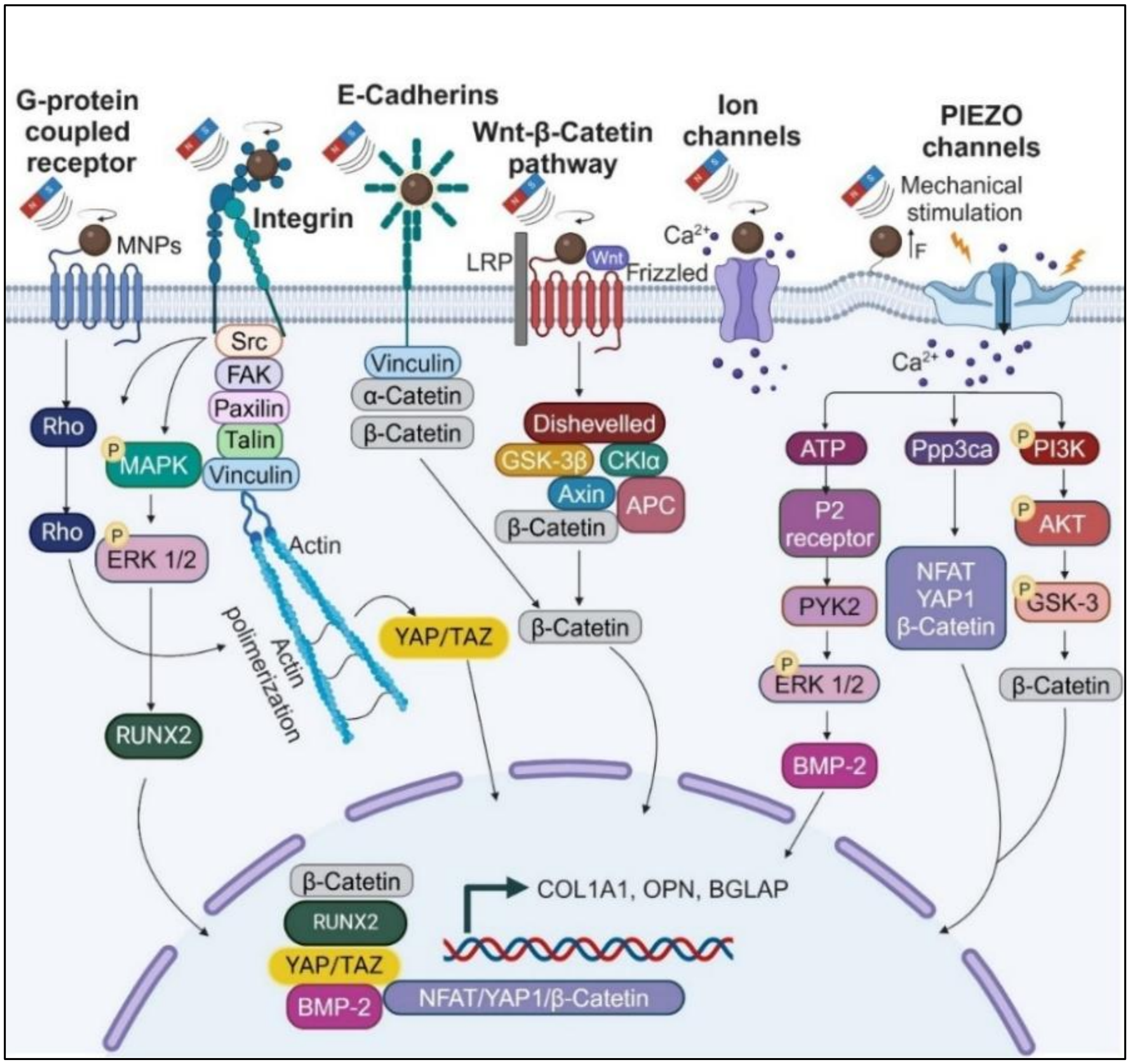

Figure S7. Illustration of the typical signaling pathways activated when magnetic NPs are attached to the cell surface and exposed to magnetic fields. Image created with BioRender and adapted from elsewhere.[3–5]

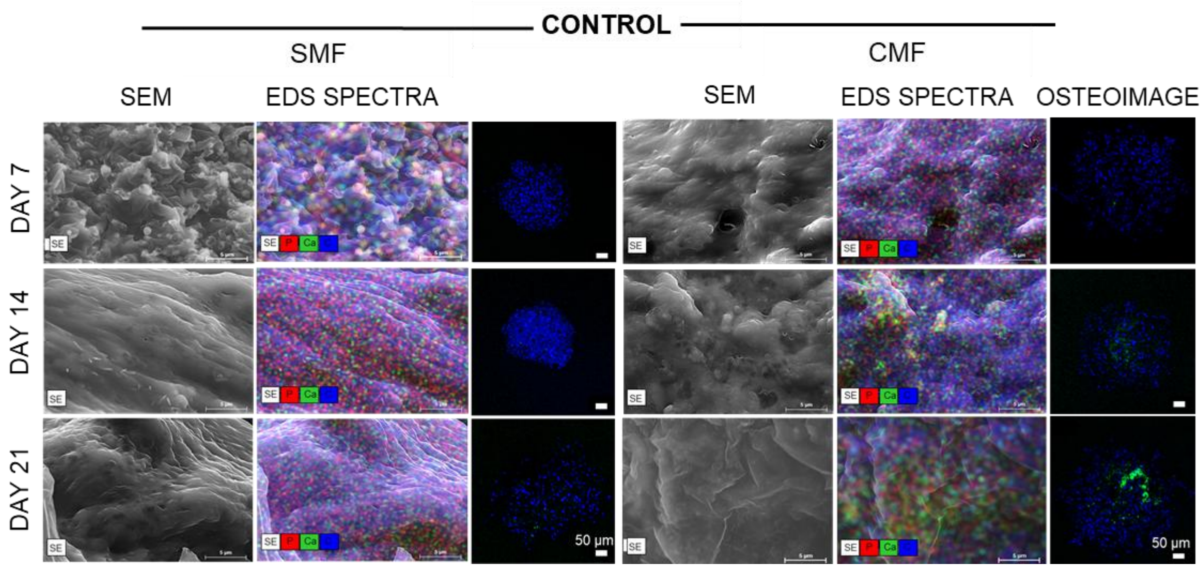

Figure S8. Mineralization of non-magnetic hASCs spheroids (control condition) under SMF and CMF conditions. Analysis was conducted using SEM, EDS and OsteoImage™ staining assay. Scale bar: 5µm for SEM and EDS micrographs; 50 µm for OsteoImage™ images.

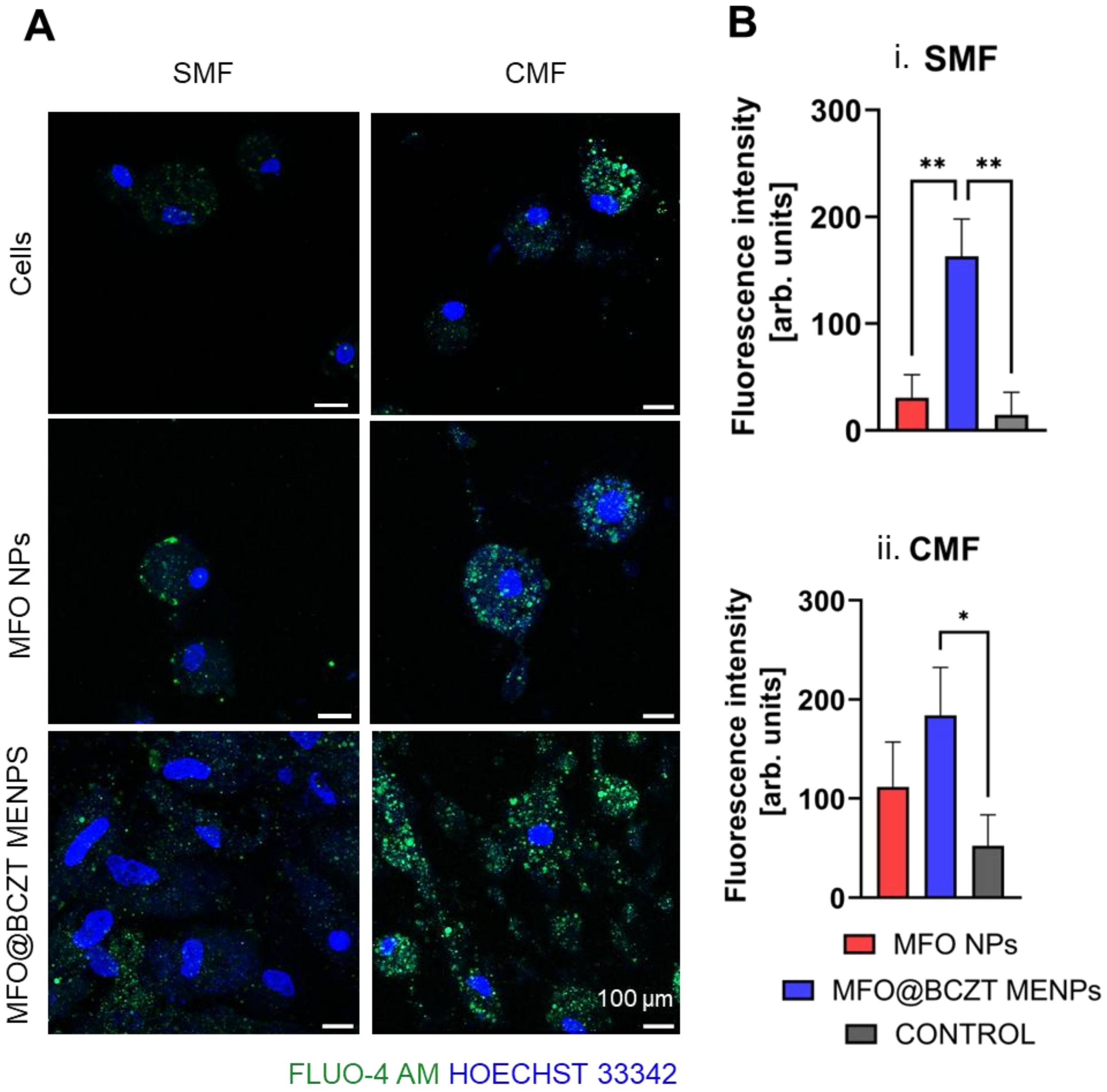

Figure S9. Dynamics of intracellular Ca^{2+} influx in magnetized and non-magnetized hASCs under overnight SMF and CMF stimulation. A) Representative confocal microscopy images showing intracellular calcium (green) labeled with the fluorescent dye Fluo-4 AM and nuclei (blue) stained with Hoechst 33342. B) Quantification of intracellular Ca^{2+} fluorescence in i) SMF and ii) CMF stimulation. Data in i) and ii) are presented as mean ± SD (*n* =3).

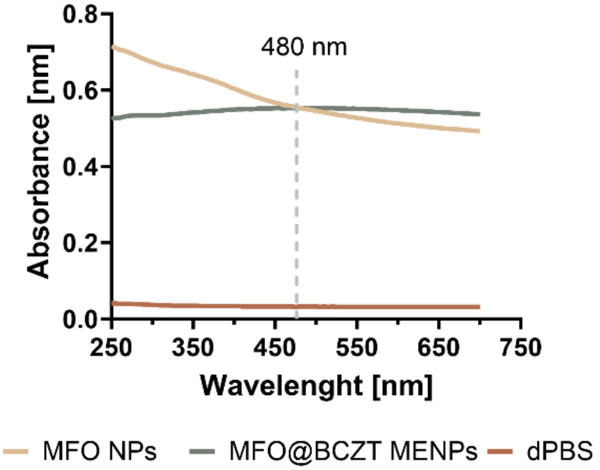

Figure S10. **UV–Vis absorbance spectra of MFO NPs and MFO@BCZT MENPs recorded between 250 and 900 nm to identify optimal wavelengths for comparative analysis.**

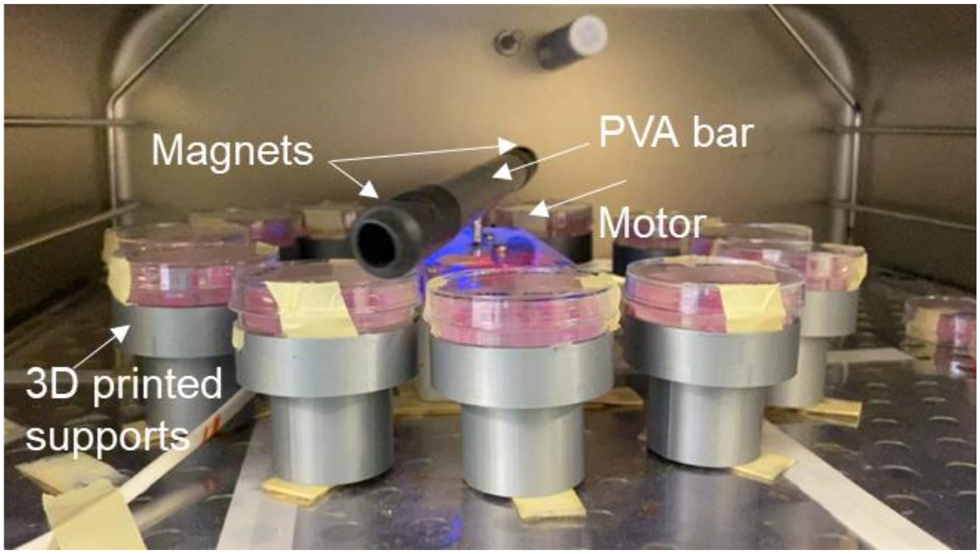

Figure S11. Photograph of the lab-made apparatus device used to generate the CMF conditions. The setup consists of a PVA bar attached to a rotating motor, with two magnets fixed at each end. Custom 3D-printed supports were used to elevate the samples to the optimal height for magnetic stimulation.

REFERENCES

(1) Vtyurina, N.; Åberg, C.; Salvati, A. Imaging of Nanoparticle Uptake and Kinetics of Intracellular Trafficking in Individual Cells. *Nanoscale* **2021**, *13* (23), 10436–10446. https://doi.org/10.1039/D1NR00901J.

(2) Donahue, N. D.; Acar, H.; Wilhelm, S. Concepts of Nanoparticle Cellular Uptake, Intracellular Trafficking, and Kinetics in Nanomedicine. *Adv Drug Deliv Rev* **2019**, *143*, 68–96. https://doi.org/10.1016/j.addr.2019.04.008.

(3) Latypova, A. A.; Yaremenko, A. V.; Pechnikova, N. A.; Minin, A. S.; Zubarev, I. V. Magnetogenetics as a Promising Tool for Controlling Cellular Signaling Pathways. *J Nanobiotechnology* **2024**, *22* (1), 327. https://doi.org/10.1186/s12951-024-02616-z.

(4) Du, Y.; Xu, B.; Li, Q.; Peng, C.; Yang, K. The Role of Mechanically Sensitive Ion Channel Piezo1 in Bone Remodeling. *Front Bioeng Biotechnol* **2024**, *12*, 01–18. https://doi.org/10.3389/fbioe.2024.1342149.

(5) Wang, J.; Sun, Y.-X.; Li, J. The Role of Mechanosensor Piezo1 in Bone Homeostasis and Mechanobiology. *Dev Biol* **2023**, *493*, 80–88. https://doi.org/10.1016/j.ydbio.2022.11.002.
